# RIP1 is a central signaling protein in regulation of TNF-α/TRAIL mediated apoptosis and necroptosis during Newcastle disease virus infection

**DOI:** 10.18632/oncotarget.17970

**Published:** 2017-05-18

**Authors:** Ying Liao, Hua-xia Wang, Xiang Mao, Hongjie Fang, Huang Wang, Yanrong Li, Yingjie Sun, Chun Meng, Lei Tan, Cuiping Song, Xusheng Qiu, Chan Ding

**Affiliations:** ^1^ Department of Avian Diseases, Shanghai Veterinary Research Institute, Chinese Academy of Agricultural Sciences, Shanghai 200241, P. R. China; ^2^ Jiangsu Co-innovation Center for Prevention and Control of Important Animal Infectious Diseases and Zoonoses, Yangzhou 225009, P. R. China; ^3^ College of Veterinary Medicine, Nanjing Agricultural University, Nanjing 210095, P. R. China

**Keywords:** NDV, apoptosis, necroptosis, RIP1

## Abstract

Newcastle disease virus (NDV) is an oncolytic virus which selectively replicates in tumor cells and exerts anti-tumor cytotoxic activity by promoting cell death. In this study, we focus on characterization of the underlying mechanisms of NDV-induced cell death in HeLa cells. We find that NDV Herts/33 strain triggers both extrinsic and intrinsic apoptosis at late infection times. The activation of NF-кB pathway and subsequent up-regulation of TNF-α/TRAIL initiates extrinsic apoptosis, leading to activation of caspase 8 and cleavage of Bid into tBid. tBid transmits the extrinsic apoptotic signals to mitochondria and mediates intrinsic apoptosis, which is hallmarked by cleavage of caspase 9. Moreover, RIP1 is cleaved into RIP1-N and RIP1-C at D324 by caspase 8, and this cleavage promotes apoptosis. Surprisingly, over expression of RIP1 reduces apoptosis and depletion of RIP1 promotes apoptosis, suggesting full length RIP1 is anti-apoptotic. Moreover, necroptosis hallmark protein MLKL is activated by phosphorylation at 12-24 h.p.i., and RIP1 regulates the level of phosphor-MLKL. Immunostaining shows that RIP1 aggregates to stress granules (SGs) at 8-24 h.p.i., and phosphor-MLKL is also recruited to SGs, instead of migrating to plasma membrane to exert its necrotic function. Immunoprecipitation study demonstrates that RIP1 bind to phosphor-MLKL, and depletion of RIP1 reduces the aggregation of MLKL to SGs, suggesting that RIP1 recruits MLKL to SGs. Altogether, NDV infection initiates extrinsic apoptosis via activation of NF-кB and secretion of TNF-α/TRAIL. Activation of caspase 8 by TNF-α/TRAIL and subsequent cleavage of Bid and RIP1 transmit the death signals to mitochondria. Meanwhile, virus subverts the host defensive necroptosis via recruiting phosphor-MLKL by RIP1 to SGs. Thus, RIP1 is a central signaling protein in regulation of apoptosis and necroptosis during NDV infection.

## INTRODUCTION

Newcastle disease virus (NDV) is highly contagious avian pathogen, which belongs to the family *Paramyxoviridae* [1, 2]. Similar to other paramyxoviruses, NDV is an enveloped virus with negative-sense single-stranded RNA, which is 15186 nucleotides in length [3]. The RNA genome encodes six structural proteins: hemagglutinin-neuraminidase (HN), fusion glycoprotein (F), matrix protein (M), nucleoprotein (NP), phosphoprotein (P), and large polymerase protein (L). HN and F are on the viral outer membrane, mediate receptor binding and membrane fusion, thereby determining the cell entry. M protein forms an inner protein layer below the inner leaflet of the viral membrane and plays essential role in virus assembly and budding. NP, P and L protein associate with the viral RNA to form the ribonucleoprotein complex (RNP), and participate in virus genome replication [4, 5]. During the transcription of the P gene, two additional non-structural proteins, V and W, are transcribed by RNA editing [6]. The V protein interferes with STAT signaling and prevents interferon stimulated genes expression, conferring NDV the ability to evade the interferon responses [7–9].

Cell death is a common host defense mechanism to eliminate virus-infected cells. Apoptosis is a major form of programmed cell death, hallmarked by characteristic morphological features including membrane blebbing, chromatin condensation, genomic DNA fragmentation, and formation of apoptotic bodies [10–12]. A variety of stimuli induced apoptosis via activation of either cell surface death receptors (extrinsic apoptosis) or mitochondria effectors (intrinsic apoptosis). The extrinsic apoptosis is triggered by the binding of death ligands (FAS ligand, TNF-α, or TRAIL) to cell surface receptors (FAS, TNFR, or death receptor 4 or 5) [13, 14]. The well characterized death receptor signaling is the binding of TNF-α to TNFR1 leads to trimerization of TNFR1 and the recruitment of TRADD, then assembles signaling complex including FADD, TRAF2, and RIP1 [15]. This signaling complex transmits signals downstream and activates transcription factor NF-κB. Another signaling complex is formed upon the internalization of TNF-α/TNFR1 complex in the cytoplasm, via recruitment of FADD and pro-caspase 8, resulting in the formation of the death inducing signaling complex [16]. This process leads to the cleavage and activation of caspase 8, initiating a caspase cascade by activating caspase 3 and caspase 7, or engaging mitochondrial apoptotic pathway by cleavage of Bid [17]. Bid is a BH3-only protein usually binding to Bcl-2 and Bax, suppressing apoptosis through sequestering Bax in the cytoplasm under physiological condition. Once Bid is cleaved by caspase 8 at Asp 60, the carboxyl terminus p15 (tBid) migrates from cytosol to mitochondrial outer membrane as an integral membrane protein, where it stimulates Bax to oligomerize and form pore [18, 19]. Bax pore permeabilizes and disrupts mitochondrial outer membrane, leads to release of cytochrome c and SMAC protein, subsequently blocking the caspase inhibitor XIAP and promoting the activation of caspase 9. Caspase 9 in turn cleaves and activates caspase 3, leading to apoptosis [20, 21].

Necroptosis is a programmed form of necrosis well known as a viral defense mechanism, characterized by membrane rupture and organelle swelling [22]. It allows cells to undergo “cellular suicide” in a caspase-independent manner with immunogenic nature. Most of the mechanistic understanding of this pathway derives from studies on TNFR1 signaling. When TNF-α binds to the receptor, if caspase 8 compromises, the signals may switch to the formation microfilament-like necrosome complex, which contains RIP1 and RIP3 [23, 24]. The pro-necrotic protein MLKL is subsequently phosphorylated by RIP1/RIP3, oligomerizes and forms pores on plasma membrane. The MLKL pores lead to expulsion of cellular contents into extracellular space and recruitment of immune cells to the damaged tissues, finally eliciting immune responses [25–28].

In NDV-infected chicken, death of chicken embryos and neurological damage in adult chicken are observed as the consequences of the apoptosis [29, 30]. In mammalian cells, NDV is shown to be a promising anticancer agent, killing tumor cells specific by inducing apoptosis [31]. It has been reported that NDV induced both extrinsic and intrinsic apoptosis, resulting in the loss of mitochondrial membrane potential, the release of cytochome c, and the activation of caspase 9 [32–34]. Elankumaran et al. detect the presence of secreted TNF-α by cells infected with various NDV strains, and the expression TRAIL is also detected at cell surface [32]. Virus entry, replication and viral proteins synthesis are required for optimal apoptosis induction [35]. Ghrici et al. report that UV-inactivated velogenic NDV (AF2240 strain) infection induces caspase 8 activation and mitochondrial transition pore opening as early as 1 and 2 h.p.i. [36]. NDV strain AF2240 M protein binds to and promotes Bax redistribution to the mitochondria [37]. HN protein has been shown to promote the expression of TRAIL in human peripheral blood mononuclear cells [38]. HN also induces up-regulation of caspases, loss of mitochondrial membrane potential, and an increased oxidative stress in chicken cells [39]. Despite above substantial study, the dominant signaling pathways involved in NDV-triggered cell death remain unclear.

Here, we show that NDV infection induces extrinsic death pathways via activation of NF-κB pathway and up-regulation of TNF-α and TRAIL in HeLa cells, a human cervical carcinoma cell line. Caspase 8 is activated subsequently cleaves Bid and RIP1, leading to apoptosis. The necroptosis hallmark, MLKL, is phosphorylated and recruited to SGs by RIP1, instead of migrating to plasma membrane. This results in suppression of necroptosis. This study provides novel insights into the underlying mechanisms by which NDV induces cell death in tumor cells.

## RESULTS

### NDV infection induces of both intrinsic and extrinsic apoptosis in tumor cells

**Figure 1:**
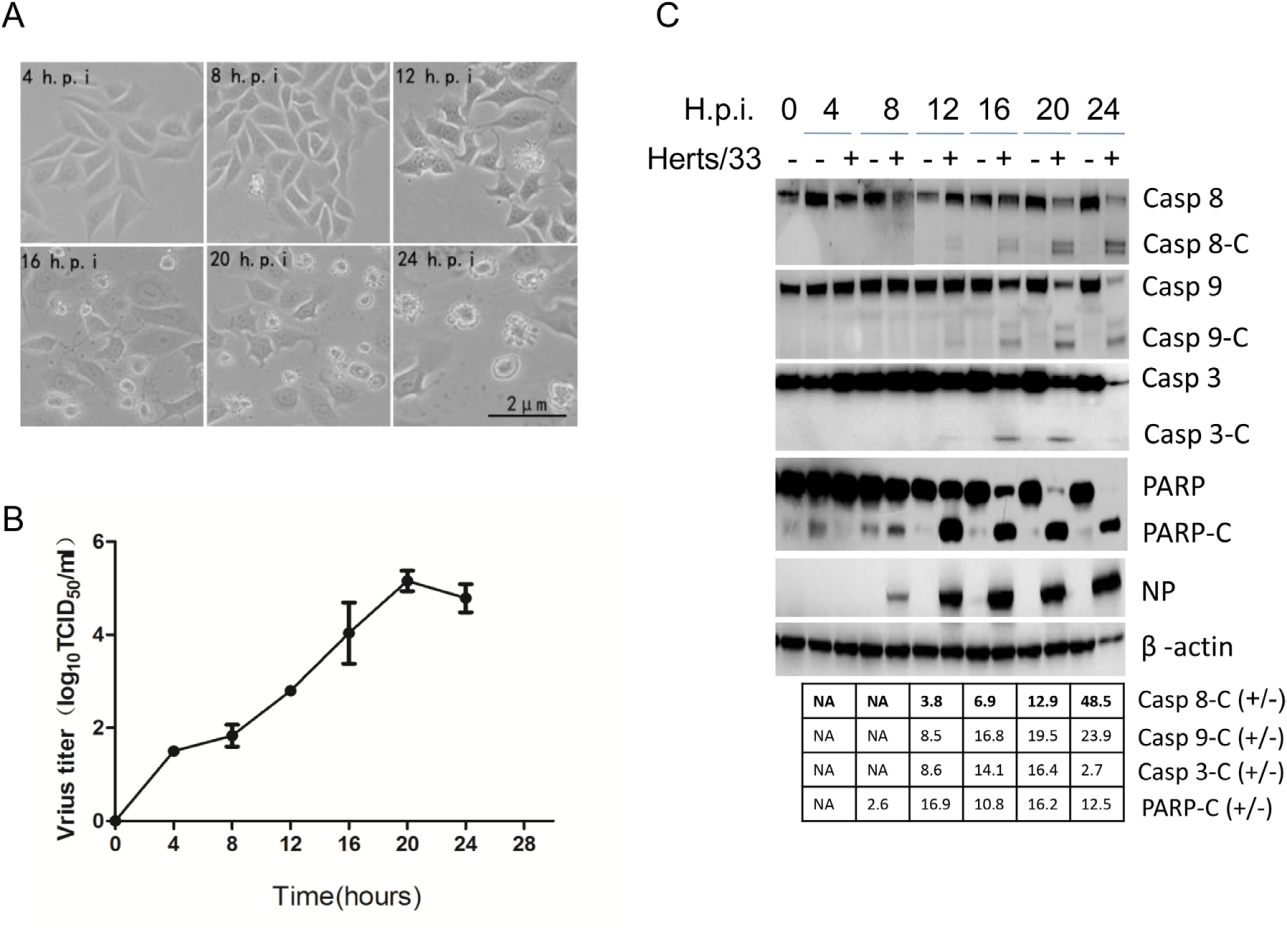
NDV infection induces both intrinsic and extrinsic apoptosis in HeLa cells **(A)** Cytopathic effects of NDV infection. HeLa cells were infected with 1 MOI of NDV. Images were taken under microscope at 4, 8, 12, 16, 20, 24 h.p.i.. **(B)** Growth curve of NDV in HeLa cells. The culture medium in Figure 1A was collected at 0, 4, 8, 12, 16, 20, and 24 h.p.i., and subjected to TCID_50_ assay using DF-1 cells. Virus growth curve was plotted based on three biological replicates, and error bars represent standard deviation of the mean (n=3). **(C)** Activation of intrinsic and extrinsic apoptosis by NDV infection. HeLa cells were mock-infected or NDV-infected, and harvested at 0, 4, 8, 12, 16, 20, and 24 h.p.i.. The NDV-induced apoptosis was analyzed with Western blot using anti-caspase 8, anti-caspase 9, anti-caspase 3, anti-PARP. NP was detected to monitor virus replication and β-actin was detected as loading control. The intensities of bands were determined by densitometry, normalized to β-actin, and shown as fold change (virus:mock).

It was reported that NDV is oncolytic through induction both extrinsic and intrinsic apoptosis [32]. To characterize the pathways that involved in NDV-induced apoptosis, we examined cytopathic effects and activation of caspases in NDV-infected HeLa cells. Cells were infected with 1 MOI of NDV, and the cytopathic effects were observed under microscope. As shown in Figure 1A, the cells membranes began blebbing at 12 h.p.i.. Along the infection time course, more cells membranes blebs were observed, and cellular morphology became irregular. The sick cells finally died and floated in the culture medium at 24 h.p.i.. To examine the virus growth curve, the cell culture medium was collected at 0, 4, 8, 12, 16, 20, and 24 h.p.i., and virus titer in the culture medium was determined with TCID_50_ assay. As shown in Figure 1B, virus particle released in the medium gradually increased along the infection time course, peaked at 20 h.p.i., and reached plateau at 20-24 h.p.i. (Figure 1B). Induction of apoptosis was subsequently analyzed with Western blot. As shown in Figure 1C, in NDV-infected cells, a 53 kDa NP band was detected from 8 to 24 h.p.i., representing active virus replication. As expected, NDV infection lead to activation of caspase 8, a cleaved fragment (18 kDa) was detected by anti-caspase 8 from 12 to 24 h.p.i.. The cleaved form (39/37 kDa) of caspase 9 was also detected at this time course, demonstrating that NDV induced apoptosis via both extrinsic and intrinsic pathway in HeLa cells. Meanwhile, an activated caspase 3 fragment (17 kDa) was detected, accompany with cleavage of PARP from full length (116 kDa) to C-terminal catalytic fragment (PARP-C, 89 kDa) (Figure 1C). To quantify the portion of cells undergoing early apoptosis and late apoptosis/necroptosis, the NDV-infected cells were stained with Annexin V-FITC and PI, and FACS (fluorescence activated cell sorting) was performed to sort the health cells, early apoptotic cells (stained with Annexin V-FITC), and late apoptotic cells/necrotic cells (stained with both Annexin V-FITC and PI). As shown in Supplementary Figure 1, 19.7% - 28.7% cells underwent early apoptosis from 16 to 20 h.p.i., only few cells underwent late apoptosis/necroptosis. Overall, above results demonstrate that the full activation of extrinsic and intrinsic apoptosis by NDV in HeLa cells.

### NDV infection up-regulates TNF-α and TRAIL transcription via NF-кB pathway

**Figure 2:**
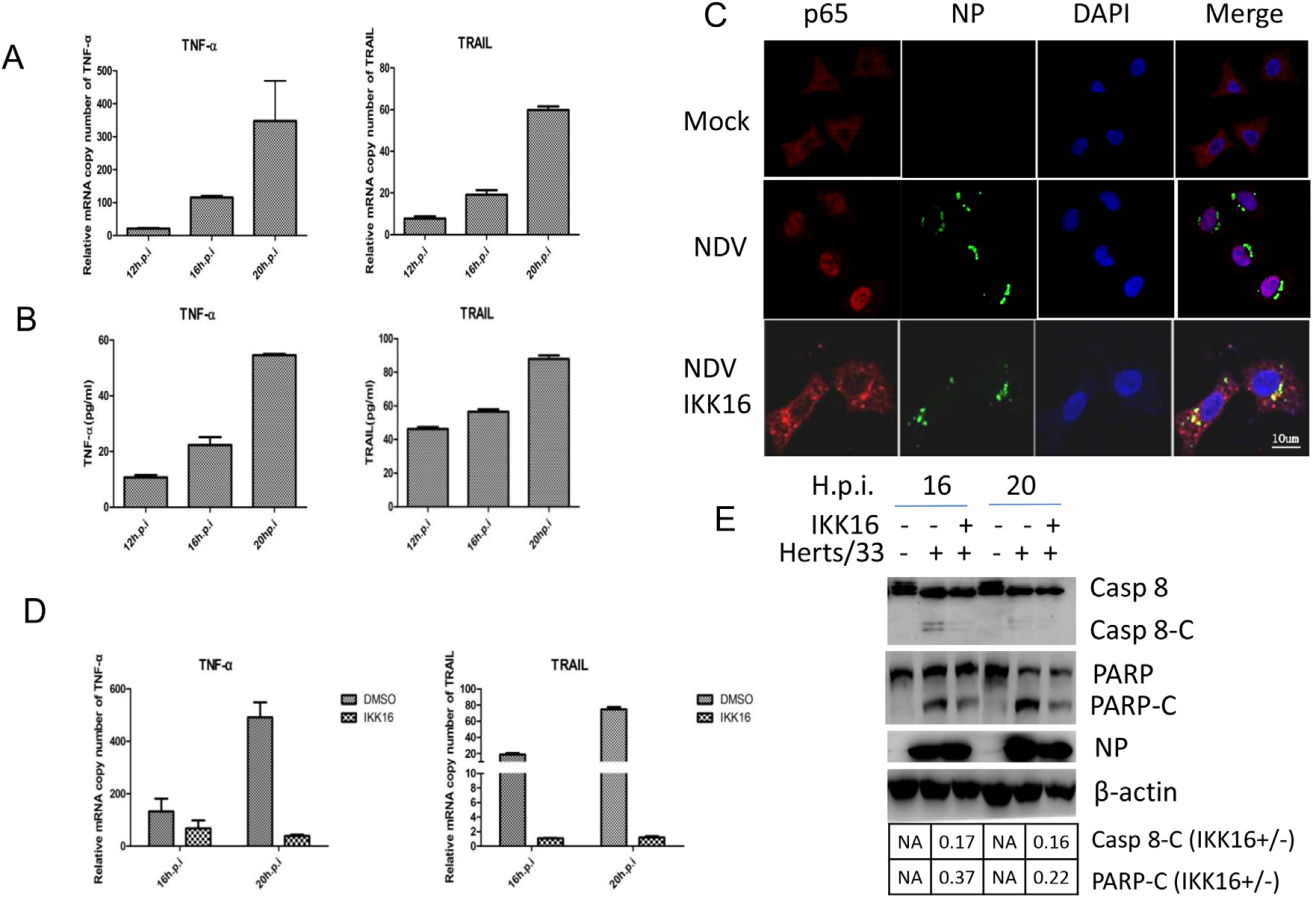
TNF-α and TRAIL are up-regulated via NF-кB pathway and promotes caspase 8 activation in NDV-infected HeLa cells **(A)** Transcriptional up-regulation of TNF-α and TRAIL during NDV infection. HeLa cells were mock-infected or NDV-infected for 12, 16, or 20 h. Total RNA was extracted and the expression levels of TNF-α and TRAIL were analyzed with semi-quantitative real time RT-PCR. The values represent means of three independent triplicate experiments ± S.D. **(B)** Secretion of TNF-α and TRAIL. The culture medium was collected at 12, 16, 20 h.p.i., and subjected to ELISA with anti-TNF-α or anti-TRAIL. The values represent means of three independent triplicate experiments ± S.D. **(C)** The nuclear translocation of p65 during NDV infection and blockage of p65 nuclear translocation by IKKβ inhibitor IKK16. HeLa cells were mock–infected or NDV-infected, followed by the treatment of DMSO or 5 μM IKK16. The translocation of p65 (red) and the expression of viral protein NP (green) were analyzed with immunostaining at 16 h.p.i. using anti-p65 and anti-NP. Nuclei were stained with DAPI (blue). Images were acquired by a META 510 confocal laser-scanning microscope (Zeiss). Merged images illustrate DAPI/NP/p65 fluorescence. **(D)** The inhibition of TNF-α and TRAIL transcription by IKK16. HeLa cells were mock-infected or NDV-infected, followed by the treatment of DMSO or 5 μM IKK16. Total RNA was extracted 16 and 20 h.p.i., and subjected to semi-quantitative real time RT-PCR using primers targeting to TNF-α and TRAIL mRNA, respectively. The values represent means of three independent triplicate experiments ± S.D. **(E)** Inhibition of apoptosis by IKK16. Cell samples in Figure 1D were lysed and analyzed by Western blot using anti-caspase 8 and anti-PARP. The intensities of bands were determined by densitometry, normalized to β-actin, and shown as fold change (IKK16+/−).

It is well known that caspase 8 is activated by the stimulation of TNF-α, FasL, or TRAIL. Subsequently, we examined whether NDV infection stimulates the expression of TNF-α, FasL, and TRAIL using semi-quantitative real time RT-PCR. As shown in Figure 2A, in NDV-infected cells, TNF-α mRNA was significantly enhanced by 23-fold, 123-fold and 314-fold at 12, 16 and 20 h.p.i., respectively, compared to that in mock-infected cells. Meanwhile, TRAIL mRNA was also up-regulated by 8-fold, 19-fold and 60-fold at 12, 16 and 20 h.p.i., respectively. Unfortunately, the detection of FasL mRNA was not successful due to unknown reason. The secretion of TNF-α and TRAIL protein was next examined by ELISA. Figure 2B showed that both proteins were successfully expressed and secreted into medium. These results demonstrate that NDV infection stimulates the expression and autocrine of pro-apoptotic factor TNF-α and TRAIL.

The transcription of TNF-α and TRAIL was usually under the control of transcription factor NF-кB. The activation of NF-кB during NDV infection was examined by checking the nuclear translocation of NF-кB subunit p65. HeLa cells were infected with NDV or mock-infected, and subjected to immunostaining at 16 h.p.i.. As shown in Figure 2C, in mock-infected cells, p65 was diffused in cytoplasm. However, in NDV-infected cells, p65 was translocated to nucleus, suggesting the activation of NF-кB. When IKKβ inhibitor IKK16 (5 μM) was incubated with NDV-infected cells, p65 was detained in the cytoplasm, confirming inhibition of NF-κB nuclear translocation by IKK16 (Figure 2C). In consistent with the suppression of NF-κB, the transcription of TNF-α and TRAIL were significantly decreased by the treatment of IKK16 (Figure 2D). This result verify that the TNF-α and TRAIL are induced via NF-кB during NDV infection. In accordance to the suppression of TNF-α and TRAIL, the cleavage of caspase 8 and PARP was also reduced to certain levels by IKK16 treatment (Figure 2E). It is noted that in IKK16-treated cells, NDV replication does not altered significantly, which is monitored by viral protein NP. To summarize, these results reveal that NDV infection induces extrinsic apoptosis by promoting the expression and secretion of TNF-α and TRAIL via NF-кB pathway.

### NDV infection activates caspase 8 and transmits signals to mitochondria via cleaving Bid to tBid

**Figure 3:**
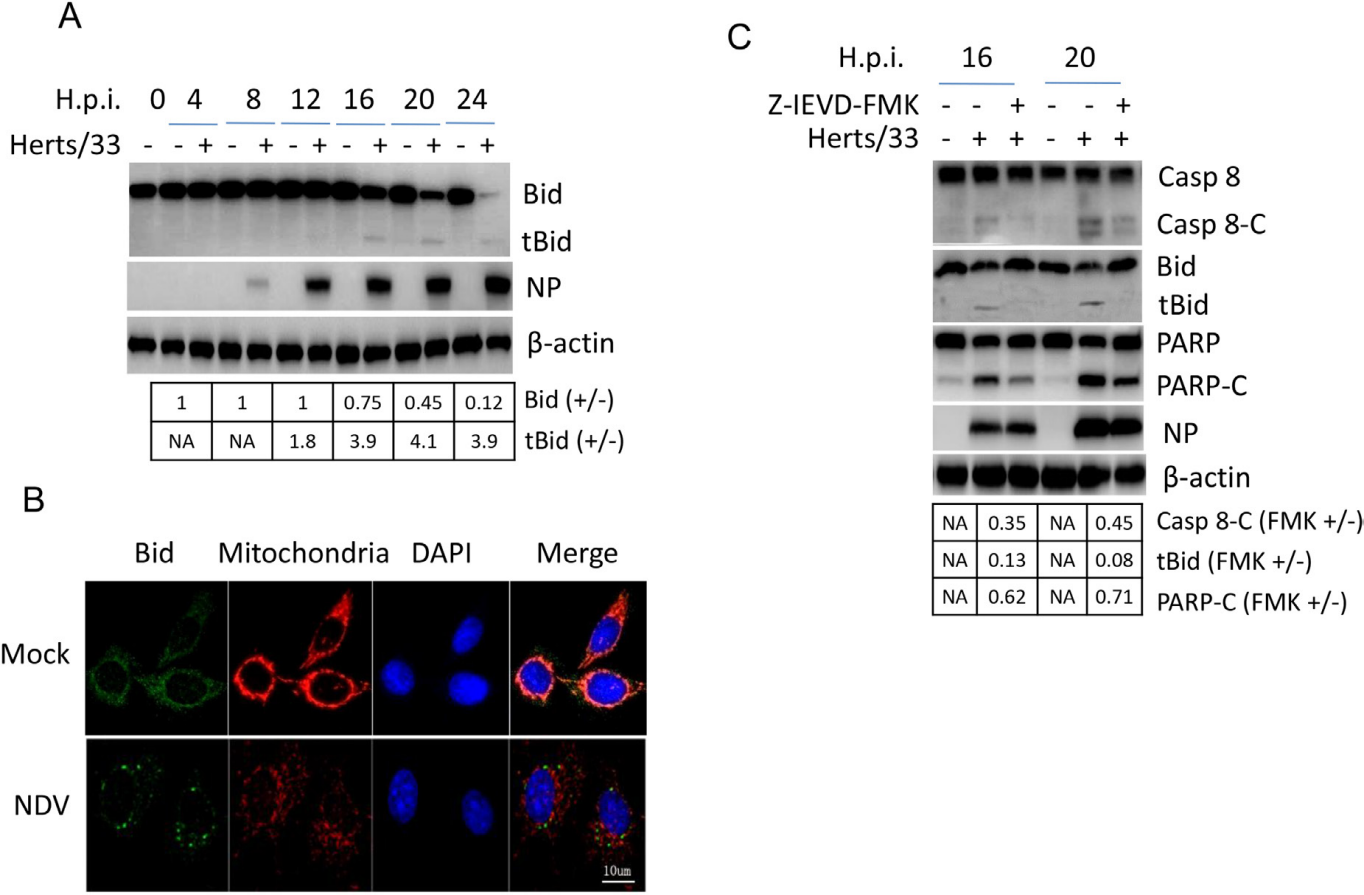
Bid is cleaved by caspase 8 and promotes apoptosis during NDV infection **(A)** The cleavage of Bid during NDV infection. Cells samples in Figure 1A were analyzed with Western blot using indicated antibodies. The intensities of Bid and tBid bands were determined by densitometry, normalized to β-actin, and shown as fold change (virus:mock). **(B)** The subcellular distribution of Bid during NDV infection. HeLa cells were mock-infected or NDV-infected. Cells were harvested at 16 h.p.i. and the subcellular distribution of Bid (green) and mitochondria (red) were analyzed with immunostaining. Nuclei were stained with DAPI (blue). Images were acquired with a META 510 confocal laser-scanning microscope (Zeiss). Merged images illustrate DAPI/Bid/mitochondrial tracker fluorescence. **(C)** Bid cleavage and PARP cleavage are dependent on caspase 8 activity. HeLa cells were mock-infected or infected with NDV, followed by the treatment of DMSO or 40 uM caspase 8 inhibitor Z-IEVD-FMK. Cells were harvested at 20, and 24 h.p.i., and the cleavage of caspase 8, Bid, and PARP were analyzed with Western blot. The intensities of bands were determined by densitometry, normalized to β-actin, and shown as fold change (FMK+/−).

Since caspase 8 is activated during infection, we next check the cleavage of BH3-only protein Bid, a Bcl-2 family pro-apoptotic protein targeted by caspase 8. As shown in Figure 3A, a full length Bid band (22 kDa) was detected from 0 to 24 h.p.i., which was decreased at 16, 20, and 24 h.p.i.. As expected, a tBid band (15 kDa) appeared from 12 to 24 h.p.i.. Immunostaining showed that both Bid and mitochondrial tracker (deep red FM) were diffused evenly in cytoplasm in mock-infected cells. Upon NDV stimulation, mitochondrial tracker formed discrete puncta in cytoplasm; Bid aggregated and formed perinuclear puncta, and was partially co-localized with mitochondrial tracker (Figure 3B). Treatment with caspase 8 inhibitor Z-IEVD-FMK blocked caspase 8 cleavage, subsequently inhibiting Bid cleavage (Figure 3C). This result verify that the cleavage of Bid is mediated by caspase 8. Eventually, PARP cleavage was significantly reduced by Z-IEVD-FMK treatment. It is noted that inhibitor treatment does not alter NDV replication. Above results suggest caspase 8 transmit extrinsic apoptotic signal to mitochondria via cleavage of Bid, thereby resulting in mitochondria mediated apoptosis.

### RIP1 is cleaved by caspase 8 at D324

**Figure 4:**
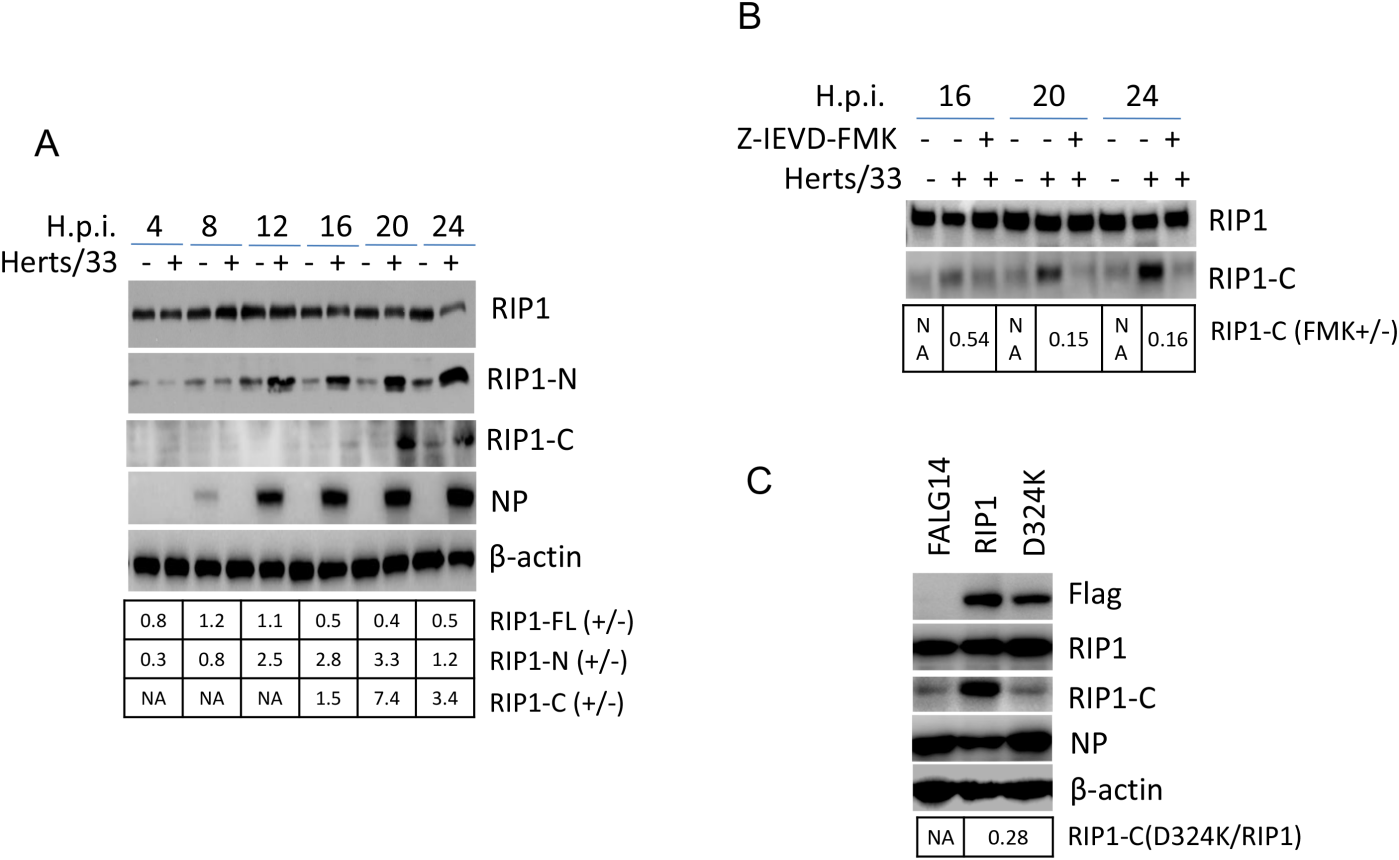
The cleavage of RIP1 at D324 by caspase 8 promotes apoptosis during NDV infection **(A)** RIP1 is cleaved during NDV infection. Cells samples in Figure 1A were analyzed with Western blot to check the cleavage of RIP1, using anti-RIP1-N or anti-RIP1-C. The intensities of bands were determined by densitometry, normalized to β-actin, and shown as fold change (virus : mock). **(B)** The cleavage of RIP1 is dependent on caspase 8 activity. HeLa cells were mock-infected or infected with NDV, followed by the treatment of DMSO or 40 μM Z-IEVD-FMK. Cells were harvested at 16, 20, and 24 h.p.i. and the cleavage of RIP1 was analyzed with Western blot using anti-RIP1-C. The intensities of RIP1 and RIP1-C bands were determined by densitometry, normalized to β-actin, and shown as fold change (FMK+/−). **(C)** RIP1 is cleaved at D324 during NDV infection. HeLa cells were transfected with Flag14, Flag14-RIP1, and Flag14-D324K for 20 h, followed by NDV infection. Cells were harvested at 20 h.p.i. and subjected to Western blot analysis to examine the expression and cleavage of RIP1. The intensities of RIP1-C bands were determined by densitometry, normalized to β-actin, and shown as fold change (D324K/RIP1).

RIP1 is another substrate targeted by caspase 8. This protein contains a N-terminal kinase domain (RIP1-N), an intermediate domain (RIP1-Z), and a C-terminal death domain (RIP-C). Cleavage of RIP1 by caspase 8 results in the blockage of TNF-induced NF-кB activation and promotes death receptor-mediated apoptosis [40]. The cleavage of RIP1 during NDV infection was examined by Western blot analysis using anti-RIP-N or anti-RIP1-C. Results showed that from 16 to 24 h.p.i., the full length RIP1 (78 kDa) was decreased, with an increase of RIP1-N (37 kDa) and RIP1-C (41 kDa) fragments (Figure 4A). These results reveal that a certain amount of RIP1 is cleaved into RIP1-N and RIP1-C at late infection times. Treatment with caspase 8 inhibitor Z-IEVD-FMK reduced the level of RIP1-C (Figure 4B), confirming that RIP1 is cleaved by caspase 8. Therefore, NDV infection lead to the cleavage of the RIP1 via activation of caspase 8.

It has been reported that RIP1 is cleaved at D324 and the C-terminal fragment promotes apoptosis [40]. To confirm the cleavage site of RIP1 in NDV infected cells, RIP1 D324 was mutated to K to abolish the cleavage site. Wild type RIP1 and mutant D324K were cloned into vector Flag14 and expressed in HeLa cells, respectively, followed by NDV infection. Western blot analysis confirmed the successful expression of Flag-tagged RIP1 and D324K. In D324K expressing cells, less RIP-C was detected than that in wild type RIP1 expressing cells (Figure 4C), confirming RIP1 is cleaved at D324 during NDV infection.

### RIP1 migrates to SGs during NDV infection

**Figure 5:**
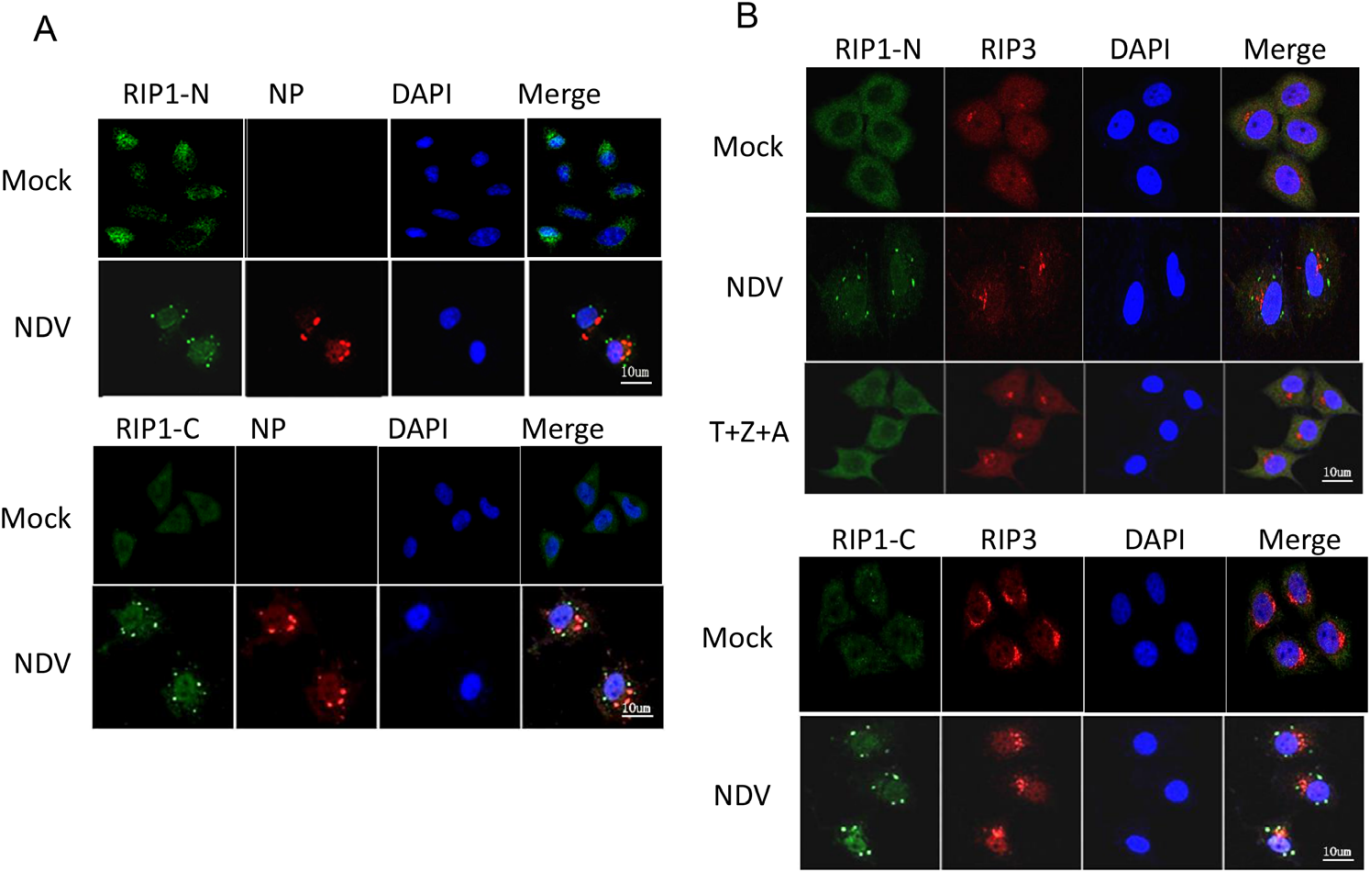

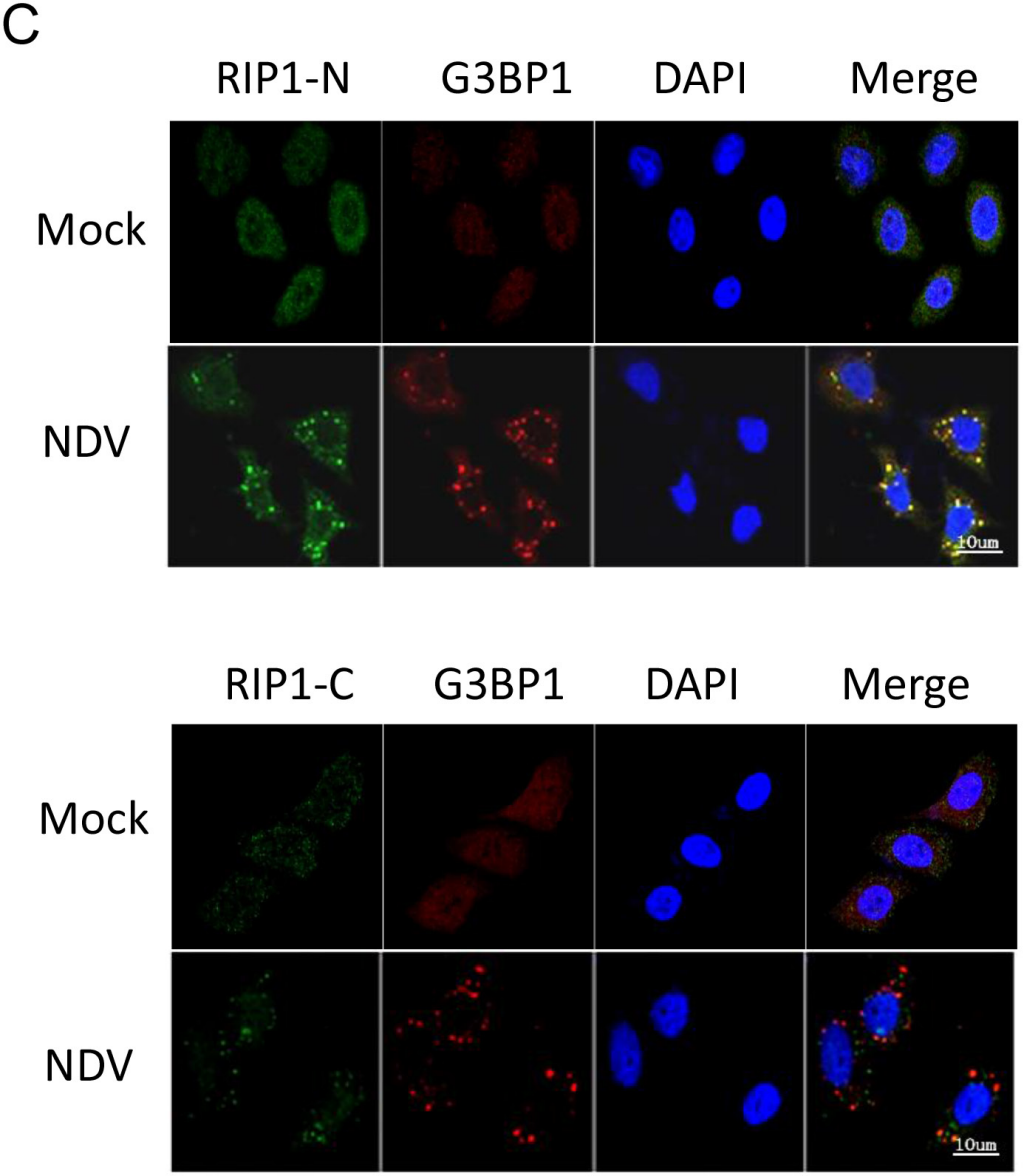
RIP1 clusteres to SGs during NDV infection **(A)** Subcellular distribution of RIP1. HeLa cells were mock-infected or infected with NDV for 16 h. The subcellular distribution of RIP1 and NP were analyzed with immunostaining using anti-RIP1-N or anti-RIP1-C (green), and anti-NP (red). Nuclei were stained with DAPI (blue). Images were acquired by a META 510 confocal laser-scanning microscope (Zeiss). Merged images illustrate RIP1/NP/DAPI fluorescence. **(B)** RIP1 is not co-localized well with RIP3 either during NDV infection or by the treatment of necroptosis stimulators. HeLa cells were mock-infected, infected with NDV for 16 h, or treated with necroptosis stimulators TNF-α, Z-VAD-FMK, and AT406 (T+Z+A) for 10 h. The subcellular distribution of RIP1 and RIP3 were analyzed with immunostaining using anti-RIP1-N or anti-RIP1-C (green), and anti-RIP3 (red). Nuclei were stained with DAPI (blue). Merged images illustrate RIP1/RIP3/DAPI fluorescence. **(C)** RIP1 is co-localized with SG marker G3BP1 during NDV infection. HeLa cells were mock-infected or NDV-infected for 16 h. The subcellular distribution of RIP1 and G3BP1 were analyzed with immunostaining using anti-RIP1-N or anti-RIP1-C (green), and anti-G3BP1 (red). Nuclei were stained with DAPI (blue). Merged images illustrate RIP1/G3BP1/DAPI fluorescence.

To explore the subcellular location of RIP1 during NDV infection, immunostaining with antibody targeting to either RIP1-N or RIP1-C was performed. Results showed that RIP1, which was uniformly diffused throughout the cytoplasm in mock-infected cells, formed discrete puncta in the cytoplasm during NDV infection (Figure 5A). It is reported that full length RIP1 interacts with RIP3 to form necrosome, phosphorylates each other, recruits MLKL and leads to its phosphorylation at Thr357 and Ser358 [27, 28, 41–44]. The formation of RIP1/RIP3 necrosome was next examined. In mock-infected cells, RIP3 was mainly diffused in the perinuclear region and the cytoplasm, with partial overlapping with RIP1. Upon NDV stimulation, RIP3 was mostly aggregated in perinuclear region as speckles (Figure 5B), and was not co-localized with the scattered RIP1 puncta. Although the speckles of RIP1 and RIP3 did not display overlapping localization, the cytoplasm diffused RIP1 and RIP3 displayed partial co-localization. Under the treatment of necroptosis stimulators T+Z+A (TNF-α, Z-VAD-FMK, AT406), RIP3 also formed speckles in the perinuclear region, however, RIP1 was remained diffused pattern in the cytoplasm, which is significantly different from that in NDV-infected cells. The cytoplasm diffused RIP1 and RIP3 co-localized well. Above results indicates the RIP1 puncta formation was virus infection specific, and a fraction of cytoplasm diffused RIP1 and RIP3 form necrosome complex.

In our study, it is found that NDV infection triggers stable formation of stress granules (SGs) [45]. We speculate the RIP1 puncta is co-localized with SGs. Immunostaining was performed to stain RIP1 and SG hallmark G3BP1 at 16 h.p.i.. As shown in Figure 5C, NDV infection induced aggregation of G3BP1 as puncta, confirming the formation of SGs. Moreover, RIP1 signals detected with anti-RIP1-N showed perfect co-localization with G3BP1, suggesting that full length RIP1 or RIP1-N migrates to SGs. However, RIP1 signals detected with anti-RIP1-C only showed partial co-localization with G3BP1, indicating the sub-cellular location of RIP-C is different from RIP1-N. Time course immunostaining showed that RIP1 and RIP1-N aggregated as speckles and were co-localized with NDV-induced SGs from 12-24 h.p.i. (Supplementary Figure 2).

### RIP1 is a central signaling protein in regulation of necroptosis and apoptosis during NDV infection

**Figure 6:**
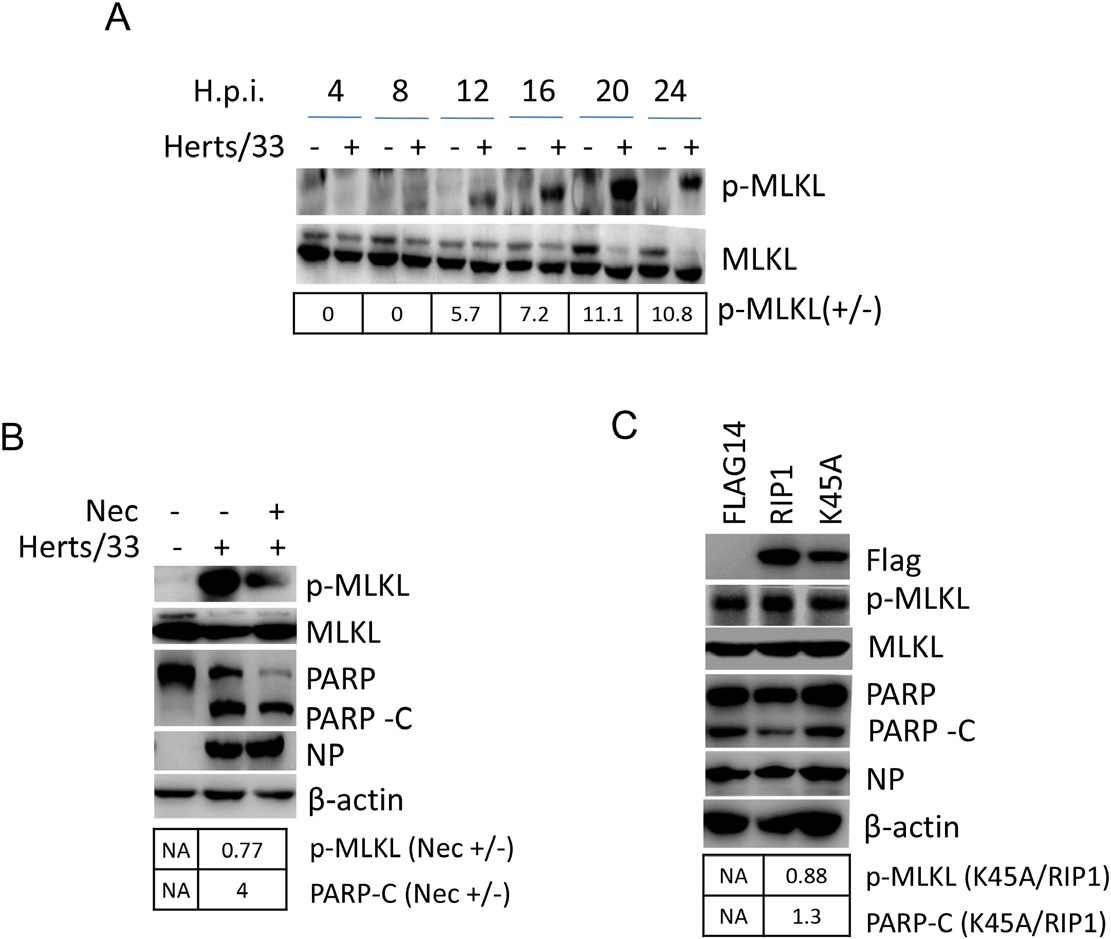

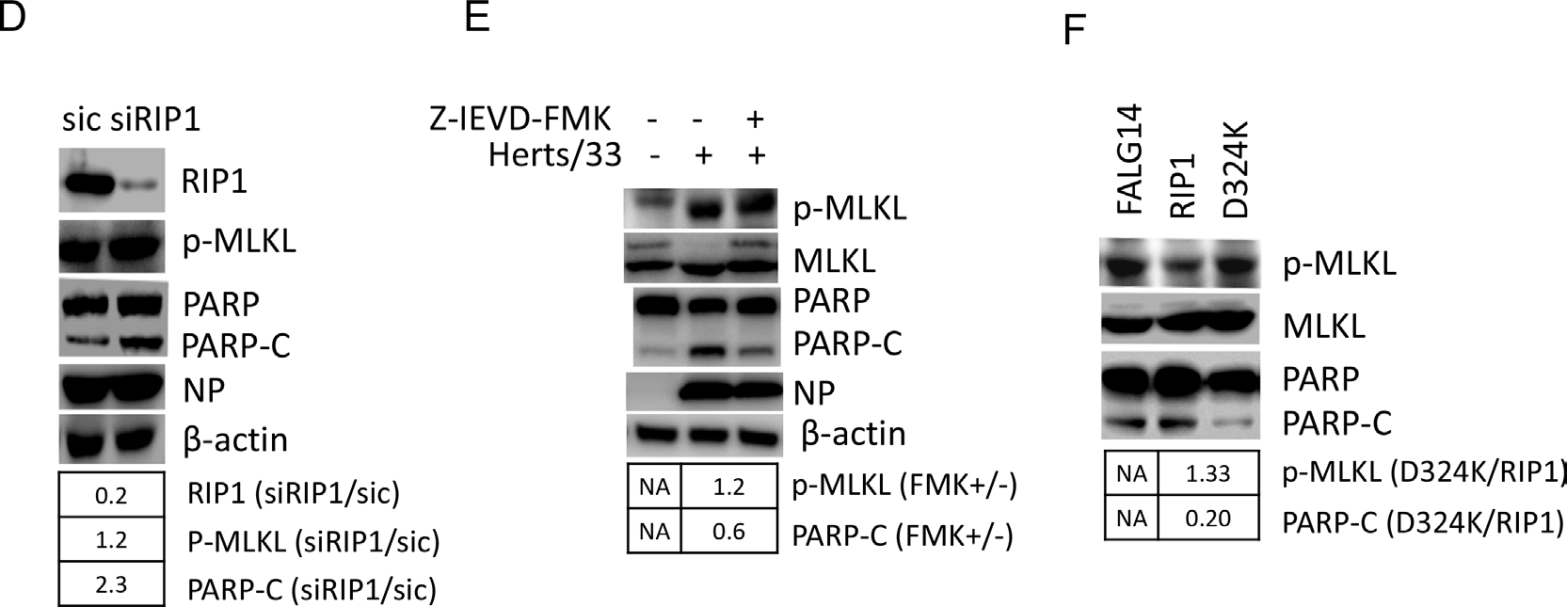
RIP1 regulates apoptosis and necroptosis during NDV infection **(A)** Phosphorylation of MLKL at Ser358. HeLa cells were mock-infected or NDV-infected. Cells were harvested at 4, 8, 12, 16, 20, and 24 h.p.i., and the phosphorylation level of MLKL at Ser358 was examined with Western blot using anti-phosphor-MLKL (Ser358). The intensities of phosphor-MLKL bands were determined by densitometry, normalized to MLKL, and shown as fold change (NDV : mock). **(B)** Inhibition of RIP1 kinase activity reduces necroptosis and promotes apoptosis. HeLa cells were infected with NDV, followed by treatment of DMSO or 200 μM RIP1 inhibitor Necrostain. Cells were harvested at 20 h.p.i., and the phosphorylation level of MLKL and cleavage of PARP were examined with Western blot. The intensities of bands were determined by densitometry. Phosphor-MLKL was normalized to MLKL, PARP-C was normalized to β-actin, and were shown as fold change (Nec +/−). **(C)** RIP1 kinase activity is anti-apoptotic. HeLa cells were transfected with Flag14, RIP1, and K45A for 20 h, followed by NDV infection. Cells were harvested at 20 h.p.i., and analyzed with Western blot. The intensities of bands were determined by densitometry. Phosphor-MLKL was normalized to MLKL, PARP-C was normalized to β-actin, and were shown as fold change (K45A/RIP1). **(D)** Depletion of RIP1 promotes apoptosis. HeLa cells were transfected with siRNA targeting to RIP1 or control siRNA (sic) for 36 h, followed by NDV infection. Cells were harvested at 20 h.p.i., and analyzed with Western blot. The intensities of bands were determined by densitometry. Phosphor-MLKL was normalized to MLKL, RIP1 and PARP-C were normalized to β-actin, and were shown as fold change (siRIP1/sic). **(E)** Inhibition of caspase 8 activity and RIP1 cleavage promotes necroptosis and reduces apoptosis. HeLa cells were mock-infected or NDV-infected, followed by treatment of DMSO or 40 μM caspase 8 inhibitor Z-IEVD-FMK. Cells were harvested at 20 h.p.i., and the phosphorylation level of MLKL and cleavage of PARP was examined by Western blot. The intensities of bands were determined by densitometry. Phosphor-MLKL was normalized to MLKL, PARP-C was normalized to β-actin, and were shown as fold change (FMK +/−). **(F)** Blockage of RIP1 cleavage by mutating D324 to K promotes necroptosis and reduces apoptosis during NDV infection. HeLa cells were transfected with Flag14, RIP1, and D324K for 20 h, followed by NDV infection. Cells were harvested at 20 h.p.i., and analyzed with Western blot. The intensities of bands were determined by densitometry. Phosphor-MLKL was normalized to MLKL, PARP-C was normalized to β-actin in Figure 4C, and were shown as fold change (D324K/RIP1).

Although RIP1 is cleaved and aggregates to SGs, a fraction of full length RIP1 may still bind to RIP3 in the cytoplasm and thereby phosphorylates MLKL, the necroptosis executor. We next examined the phosphorylation level of MLKL with Western blot using anti-phosphor-MLKL. As shown in Figure 6A, in NDV-infected cells, a 54 kDa phosphor-MLKL was detected at 12, 16, 20, and 24 h.p.i., compared to that in mock-infected cells. Total MLKL was detected as two bands in mock-infected cells, however, the upper band was reduced at 16, 20, and 24 h.p.i. during NDV infection. Above results confirm the activation of necrotic signaling during NDV infection.

Next, we investigated the role of RIP1 kinase activity in regulation of NDV-induced necroptosis and apoptosis. The NDV-infected cells were treated with necrostatin, which specifically inhibits RIP1 kinase activity. Cells were harvested at 20 h.p.i., and the phosphorylation level of MLKL and cleavage of PARP were examined with Western blot. Figure 6B showed that treatment with necrostatin resulted in low level of phosphor-MLKL, compared to that in DMSO-treated cells, demonstrating the promotion role of RIP1 kinase activity in MLKL phosphorylation. Moreover, the cleavage of PARP was increased in the presence of this inhibitor (Figure 6B), suggesting that inhibition of RIP1 kinase activity promotes apoptosis. We further cloned the RIP1 kinase-dead mutant K45A to examine the role of kinase activity in regulation necroptosis and apoptosis during NDV infection. HeLa cells were transfected with vector Flag14, wild type RIP1, or kinase-dead mutant K45A, followed by NDV infection. Western blot results confirmed the successful expression of RIP1 and K45A (Figure 6C). Compared to that in RIP1 expressing cells, less phosphor-MLKL and more PARP cleavage was observed in K45A expressing cells (Figure 6C). Meanwhile, less PARP cleavage was observed in RIP1 expressing cells, comparing to that in vector-tranfected cells (Figure 6C). Above results demonstrates that RIP1 kinase activity is pro-necrotic and anti-apoptotic. We further examined the role RIP1 in apoptosis and necroptosis by depletion of RIP1. Western blot analysis showed the successful knock down of RIP1 by siRNA (Figure 6D). The level of phosphor-MLKL was comparable in RIP1 depletion cells and control cells, however, there was more PARP cleavage in RIP1 depletion cells than that in control cells (Figure 6D). This result further confirms that RIP1 performs anti-apoptotic function in NDV-infected cells.

The role of RIP1 cleavage in regulation of apoptosis and necroptosis was next examined. Pharmacological inhibition of RIP1 cleavage via incubating caspase 8 inhibitor Z-IEVD-FMK with NDV-infected cells was performed. Western blot result showed that higher level of phosphor-MLKL and less level of PARP cleavage were observed in Z-IEVD-FMK-treated cells, compared to those in DMSO-treated cells (Figure 6E). It was noted that inhibitor treatment recovered the upper band of MLKL, which disappeared upon NDV infection. This indicates the caspase 8 activity is involved in cleavage of MLKL upper during NDV infection. The identity and function of this MLKL upper band has not been clarified yet. In consistent with above observation, exogenous expression of D324K also resulted in higher level of phosphor-MLKL and less level of PARP cleavage, compared to those in RIP1 expressing cells (Figure 6F). This results confirm that the cleavage of RIP1 by caspase 8 is pro-apoptotic and anti-necrotic during NDV infection. In conclusion, above study reveals that RIP1 kinase activity is anti-apoptotic, whereas cleavage of RIP1 at D324 is pro-apoptotic and anti-necrotic.

### Phosphor-MLKL aggregates to SGs instead of plasma membrane translocation

**Figure 7:**
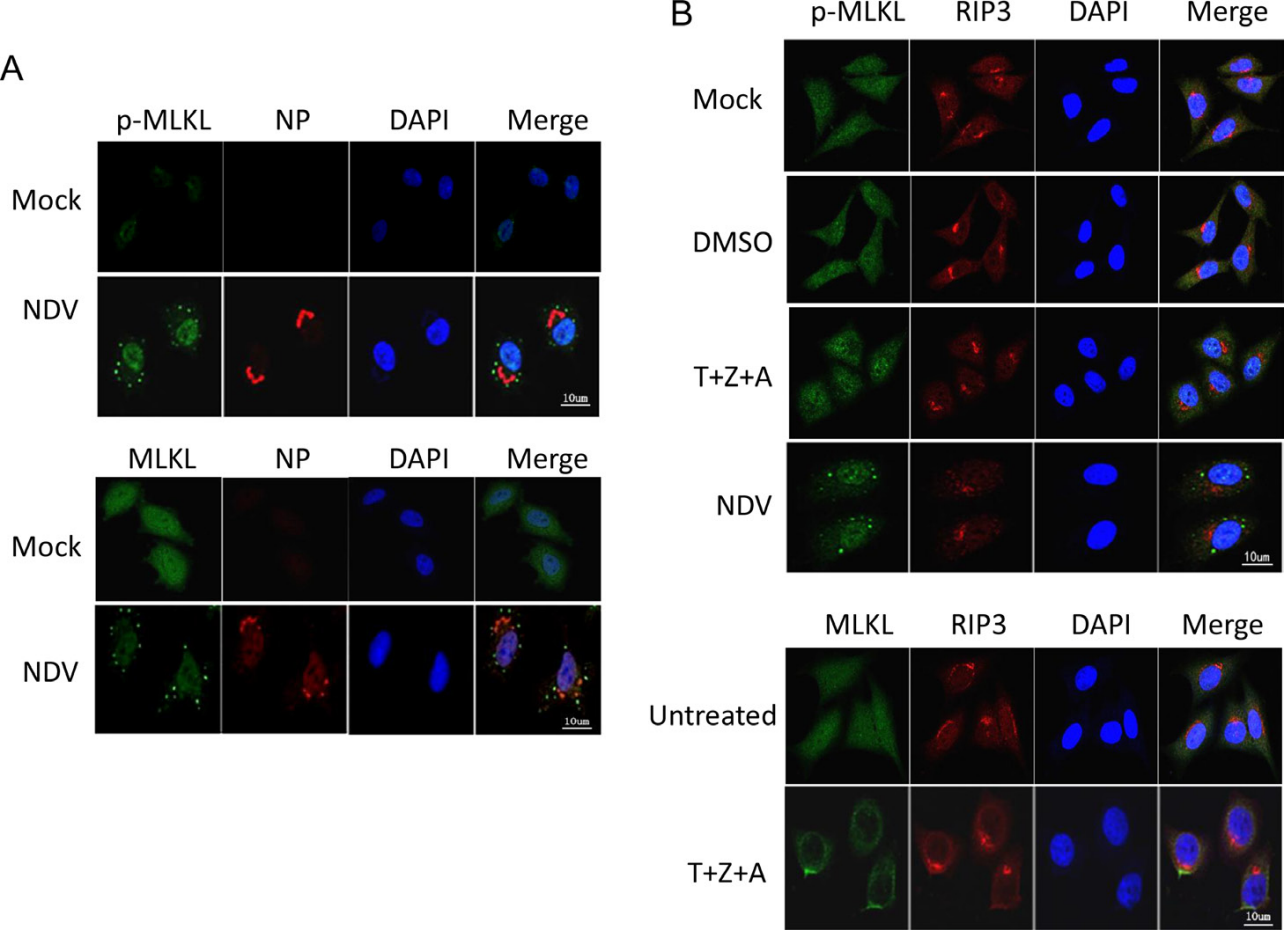

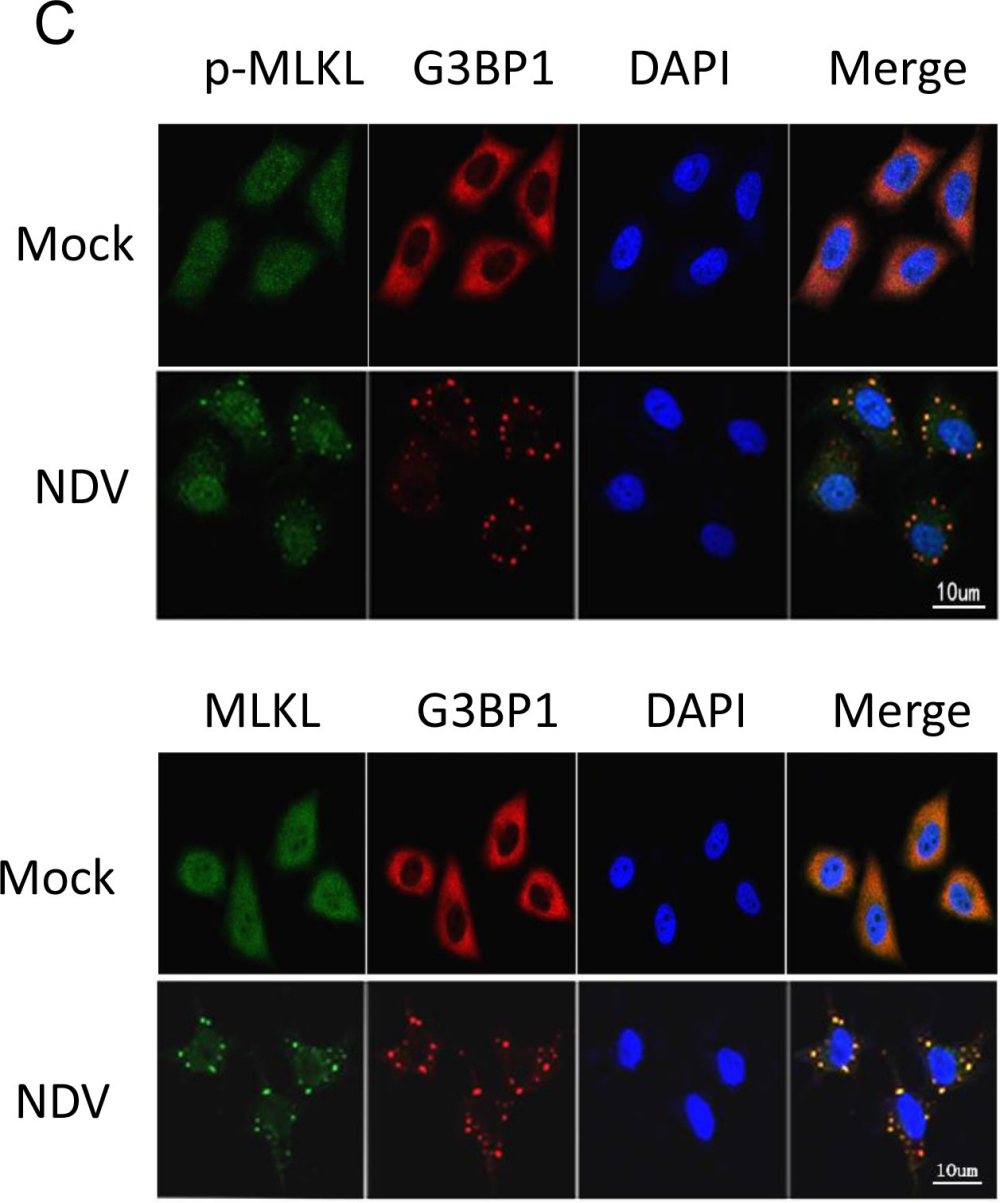
MLKL clusteres to SGs during NDV infection, instead of plasma membrane translocation **(A)** MLKL aggregates to punctate structure in the cytoplasm during NDV infection. HeLa cells were mock-infected or NDV-infected for 16 h. The subcellular distribution of phosphor-MLKL or total MLKL (green), and NP (red) were examined with immunostaining. Nuclei were stained with DAPI (blue). Merged images illustrate p-MLKL/NP/DAPI or MLKL/NP/DAPI fluorescence. **(B)** MLKL moves to plasma membrane by treatment of necroptosis stimulators (T+Z+A). HeLa cells were treated with necroptosis stimulators TNF-α, Z-VAD-FMK, and AT406 (T+Z+A) for 10 h. The subcellular distribution of phosphor-MLKL or MLKL (green), and RIP3 (red) were examined with immunostaining. Nuclei were stained with DAPI (blue). Merged images illustrate MLKL/RIP3/DAPI fluorescence. **(C)** MLKL is co-localized with SGs hallmark G3BP1 during NDV infection. HeLa cells were mock-infected or NDV-infected for 16 h. The subcellular distribution of phosphor-MLKL or MLKL (green), and G3BP1 (red) were examined with immunostaining. Nuclei were stained with DAPI (blue). Merged images illustrate MLKL/G3BP1/DAPI fluorescence.

The subcellular distribution of MLKL during NDV infection were examined by immunostaining using either anti-phosphor-MLKL or anti-MLKL. Figure 7A showed that in mock-infected cells, MLKL was diffused in nucleus and cytoplasm. However, in NDV-infected cells, cytoplasmic MLKL aggregated to punctate structure. This result is out of our expectation, as all previous reports show that phosphor-MLKL migrates to plasma membrane and forms pores, thereby mediating the release of cell content. To examine the subcellular distribution of MLKL during necroptosis, the necroptosis stimulators (T+Z+A) were incubated with cells to initiate the necroptosis. Figure 7B showed that upon the stimulation of stimulators, MLKL migrated to plasma membrane as expectation, and RIP3 was in the perinuclear region. Therefore, the formation of punctate structure of MLKL in the cytoplasm is specifically induced by NDV infection. We next investigated whether MLKL puncta was co-localized with SGs. As Figure 7C showed, during NDV infection, MLKL was really co-localized with SG marker G3BP1. Time course immunostaining showed that phosphor-MLKL aggregated to SGs from 8 to 24 h.p.i. (Supplementary Figure 3). Above results demonstrate that similar to RIP1, MLKL aggregate to SGs during NDV infection.

### Interaction between RIP1 and MLKL determines the SGs location of MLKL

**Figure 8:**
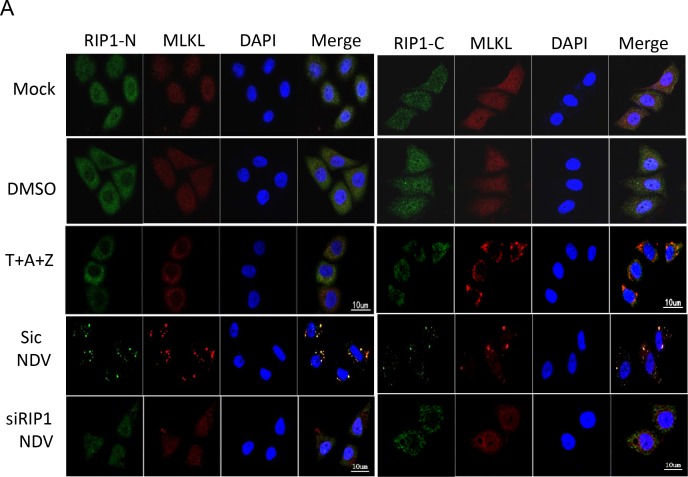

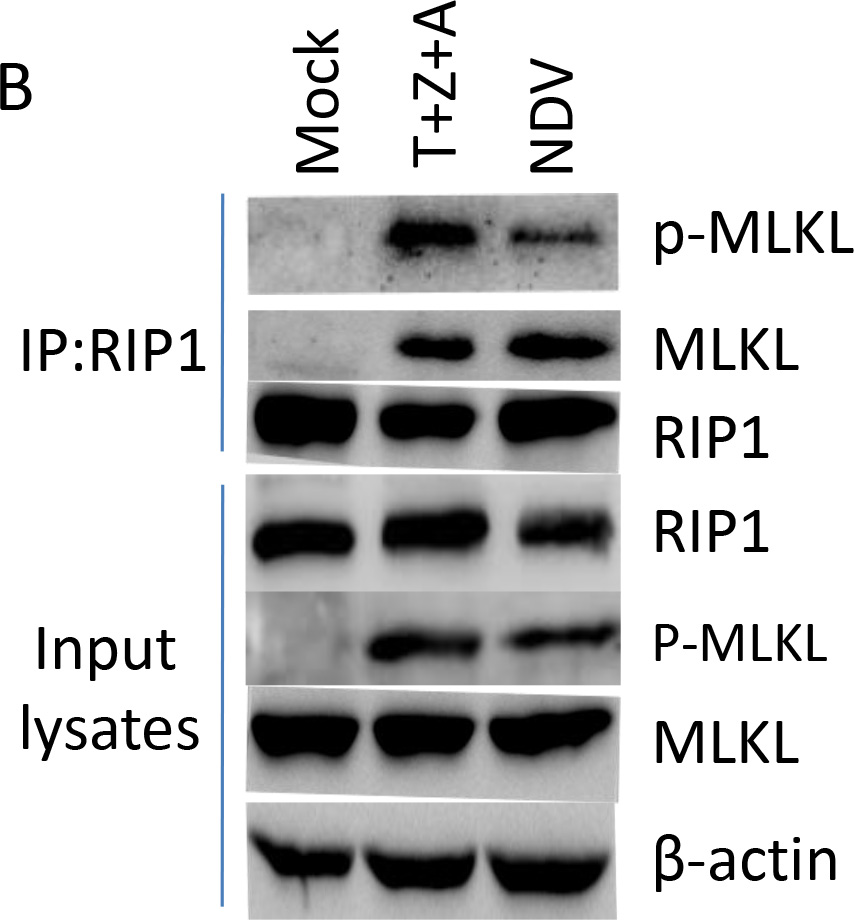
RIP1 binds to MLKL and recruits MLKL to SGs **(A)** Depletion of RIP1 prevents MLKL aggregate to SGs. HeLa cells were transfected with non-target siRNA or RIP1 siRNA for 36 h, followed with NDV infection for 16 h. Mock-infected cells, DMSO, T+A+Z stimulated cells were included as control. The subcellular distribution of RIP1 (green) and MLKL (red) was examined with immunostaining with anti-RIP1-N, or anti-RIP1-C, and anti-MLKL. Nuclei were stained with DAPI (blue). Merged images illustrate RIP1-N/MLKL/DAPI or RIP1-C/MLKL/DAPI fluorescence. **(B)** RIP1 binds to MLKL upon T+Z+A stimulation or NDV infection. HeLa cells were mock-infected, NDV-infected for 16 h, or stimulated with T+Z+A for 10 h, and subjected to immunoprecipitation using anti-RIP1-N. The pull down proteins and whole cell lysates were analyzed with Western blot.

As both RIP1 and MLKL migrate to SGs during NDV infection, we next to ask whether RIP1 recruits MLKL to SGs. siRNA knock down was performed to deplete RIP1 in HeLa cells, followed by NDV infection. Immunostaining in Figure 8A showed that in mock-infected cells, both RIP1 and MLKL diffused in the cytoplasm and nuclear. When cells were stimulated with T+Z+A, RIP1 and MLKL moved to plasma membrane. In NDV-infected non-target siRNA transfected cells, RIP1-N and MLKL were co-localized well in punctate structure, RIP1-C and MLKL displayed partial co-localization. However, in RIP1 depletion cells, less puncta of RIP1 and MLKL were observed, demonstrating the punctate aggregation of MLKL is dependent on RIP1. Above finding is unexpected, suggesting that NDV employs RIP1 to recruit MLKL in SGs.

We next ask whether there is direct binding between RIP1 and MLKL during NDV infection. HeLa cells were mock-infected, treated with necroptosis stimulators T+Z+A, or NDV-infected, followed with immunoprecipitation using anti-RIP1. Western blot analysis with anti-RIP1-N showed the successful pull down of RIP1. Both necroptosis stimulator-treated cells and NDV-infected cells showed successful pull down of phosphor-MLKL together with RIP1. As expected, in mock-infection cells, no MLKL was pull down together with RIP1. These results demonstrate the direct binding between these two proteins. Collectively, above findings demonstrate that although RIP1 is involved in regulation of phosphor-MLKL, however, it recruits MLKL to SGs. Therefore, MLKL does not really move to plasma membrane to exert its necrotic function. This observation was further confirmed by FACS experiment, which showed that few cells undergoing necroptosis, and apoptosis is the dominant cell death pathway (Supplementary Figures 1 and 4).

## DISCUSSION

Both extrinsic apoptosis and necroptosis are activated by the TNF family death receptor and TRAIL receptor. The most extensively studied extrinsic apoptotic pathway and necroptotic pathway is mediated by TNF-α-TNFR1 signaling. NDV is an oncolytic virus killing tumor cells via selective replication in tumor cells and induction of cells death. Apoptosis has been identified as a major hallmark of NDV-mediated cell death and mitochondrial apoptotic pathway has been identified as the dominant pathway. Necroptosis is an alternative form of programmed death, which has been identified as a part of cellular response to NDV infection in glioblastoma, marked by cellular swelling, extensive membrane and cell disintegration and karyolysis [46]. However, the signalings that NDV induces the extrinsic apoptotic pathway, and whether necroptosis contributes to NDV-induced cell death, have not been well resolved yet.

**Figure 9:**
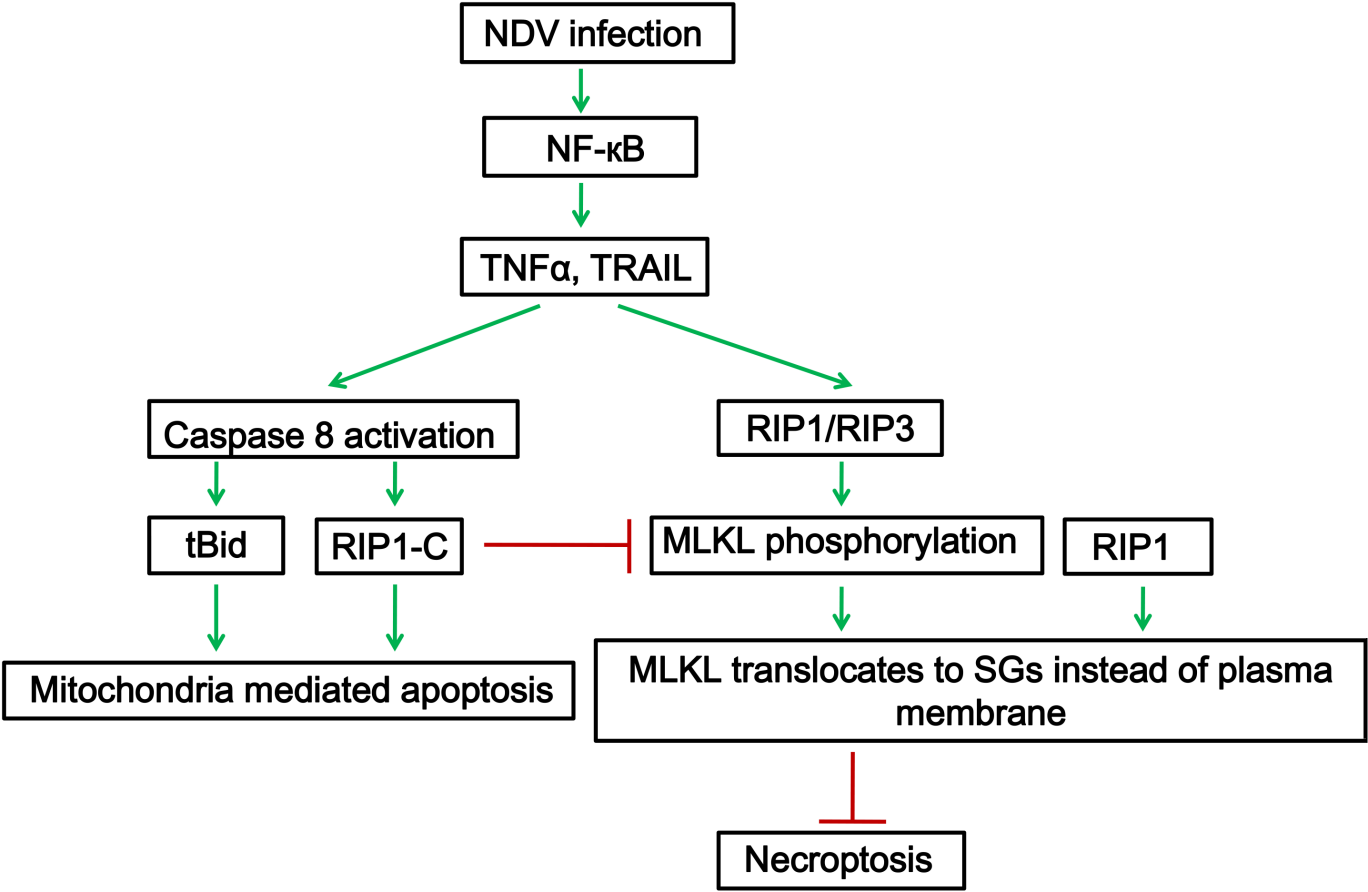
RIP1 is a central signaling protein in regulation of TNF-α/TRAIL mediated extrinsic apoptosis and necroptosis during NDV infection NDV infection activates NF-κB, induces the expression and secretion of TNF-α and TRAIL. TNF-α and TRAIL binds to TNF receptor or TRAIL receptor on cell surface, and the pro-apoptotic complex is formed. Caspase 8 is activated and cleaves Bid and RIP1, promotes apoptosis. Meanwhile, full length RIP1 promotes the phosphorylation of MLKL. RIP1 binds to phosphor-MLKL and recruits it to SGs, prevents it move to plasma membrane, thereby inhibiting necroptosis.

Here, we find that NDV infection activates NF-кB pathway and induces the transcription and secretion of TNF-α and TRAIL. TNF-α and TRAIL are involved in killing neighbor cells, via binding to receptors on neighbor cells. Caspase 8 is activated and lead to cleavage of Bid, transmitts death signals to mitochondria. Moreover, caspase 8 promotes apoptosis via cutting full length RIP1 into RIP1-N and RIP1-C at D324. Although RIP1 is cleaved into two fragments, a certain amount of full length RIP1 is still detected. Out of our expectation, RIP1 aggregates to NDV-induced SGs. SGs usually are induced to sequester cytoplasmic mRNA, protein translation factors, and RNA binding proteins, thereby arresting protein translation and reliefing ER stress. We find lots of TNF-α downstream signaling proteins move to NDV-induced SGs, such as TRAF2, A20, IKKα/β, IKKγ (Unpublished data). Thus, SGs may serve as a signaling transmitting platform during NDV infection. RIP1 is involved in phosphorylation of necroptosis hallmark MLKL. Phosphor-MLKL moved to SGs by binding with RIP1, instead of moving to plasma membrane to exert the necrotic function. This suggests that NDV has evolved a mechanism to counteract the necroptosis response through recruiting the phosphor-MLKL to the SGs. The working model is summarized in Figure 9.

As necroptosis is an antiviral host defense to restrict virus replication, therefore, NDV may subvert host defense via suppressing necroptosis. This is not the first report about the subversion of necroptosis by virus. The most well studied example is Hepes simplex virus (HSV), which prevents the formation of RIP1-RIP3 complex in human cells by viral protein R1 RHIM domains [47–49]. Murine cytomegalovirus M45 also encodes RHIM competitor vIRA and block RHIM dependent homotypic interaction between RIP1, RIP3, DAI, and TRIF, thereby suppressing necroptosis [50–52]. Human cytomegalovirus IE1 protein prevents necroptosis at a step that follows RIP3 phosphorylation and activation of MLKL [53]. The suppression of necroptosis may represent a common strategy that virus used to circumvents the host defense.

In summary, this report provides a comprehensive understand of NDV-induced extrinsic apoptosis and necroptosis. Further understanding of the precise mechanisms and biological functions of SGs-localized signaling proteins will provide new insight into virus-cell interaction.

## MATERIALS AND METHODS

### Cell culture and virus

HeLa human cervical cancer cells and the DF-1 chicken fibroblast cells were purchased from American Type Culture Collection (ATCC, Manassas, VA, USA) and maintained in Dulbecco's modified Eagle's medium (DMEM) supplemented with 10% fetal bovine serum (FBS) (Hyclone, UT, USA), penicillin (100 units/ml) and streptomycin (100 μg/ml) (Invitrogen, USA) at 37°C in a 5% CO_2_ incubator.

The NDV strain Herts/33 was purchased from China Institute of Veterinary Drug Control (Beijing, China). The viruses were propagated in embryonated chicken eggs and titrated on DF-1 cells. Virus was used for infection at multiplicity of infection (MOI) of 1 throughout this study.

### Reagents and antibodies

RNA extraction reagent Trizol^®^ and Lipofectamine 3000 were purchased from Invitrogen Thermo Fisher Scientific (USA). RIP1 inhibitor necrostatin (S8037), caspase inhibitor Z-VAD-FMK (s7023), and AT406 (s2754) were purchased from Selleck Chemicals. Caspase 8 inhibitor Z-IEVD-FMK (FMK007) was purchased from R&D Systems, Inc. TNF-α (#8902) was purchased from Cell Signaling Technology. Dimethyl sulfoside (DMSO) was purchased from Sigma-Aldrch.

Monoclonal NDV NP protein antibody was raised in mice using bacterially-expressed His-tagged NP protein as the immunogen. Antibodies against caspase 8 (#9746), caspase 3 (#9665), caspase 9 (#9508), PARP (#9532), Bid (#2002), Bax (#5023), p65 (#8242), RIP1-N (#3493), RIP1-C (#4926), phosphor-MLKL (#91689), MLKL (#14993) were purchased from Cell Signaling Technology (USA). Anti-G3BP1 (ab56574) was purchased from Abcam (UK). Anti-RIP3 (sc-374639) was purchased from Santa Cruz Biotechnology (USA). Anti-β-actin (A1978) was purchased from Sigma-Aldrich (USA). The secondary IgG conjugated with HRP, FITC, or TRITC were obtained from DAKO (Denmark).

### Plasmid DNA transfection and siRNA

Plasmid DNA was transfected using lipofectamine 3000 (Invitrogen, USA). Briefly, cells were grown on 6 well plates at 70-80% confluence before transfection. Each transfection was carried out using Lipofectamine 3000 reagent according to the manufacturer's standard protocol. Cells were then infected with NDV at MOI of 1 at 24 h post-transfection and incubated for 16 h.

### Western blot analysis

Cells were lysed with 1x SDS loading buffer in the presence of 100 mM dithiothreitol and denatured at 100°C for 5 min. Equivalent amounts of protein were separated by SDS-PAGE, followed by transfer onto polyvinylidene difluoride membranes (Bio-Rad Laboratories, USA) by electroblotting. Immunoblot analysis was then performed by incubating membranes with appropriate antibodies in blocking buffer for 1 h at room temperature. After washing three times with PBST, membranes were incubated with HRP-conjugated secondary antibody for 1 h and washed with PBST thrice. Blots were developed with an enhanced chemiluminescence (ECL) detection system (GE Healthcare Life Sciences, USA) and exposed to X-ray film (Fuji, Japan). Membranes were stripped with stripping buffer (10 mM β-mercaptoethanol, 2% SDS, 62.5 mM Tris, pH 6.8) at 55°C for 30 min before re-probing with other antibodies.

### Immunostaining

HeLa cells were seeded on 4-well chamber slides and infected with NDV at MOI of 1. Cells were fixed with 4% paraformaldehyde for 10 min, washed thrice with PBS, permeabilized with 0.2% Triton X-100 for 10 min, and washed thrice with PBS. Cells were then incubated with antibody (1:200) diluted in PBS (5% BSA) for 2 h, washed thrice with PBS, and then incubated with secondary antibody conjugating with FITC or TRITC (DAKO) for 2 h (1:200 diluted in PBS, 5% BSA), followed by PBS washing. Cells were next incubated with 0.1 μg/ml DAPI diluted in PBS for 10 min and rinsed with PBS. Finally, the specimen was mounted with glass cover slips using fluorescent mounting medium (DAKO) containing 15 mM NaN_3_. Images were collected with a META 510 confocal laser-scanning microscope (Zeiss).

MitoTracker^®^ Deep Red FM (Cell signaling technology #8778) was diluted directly in DMEM to a final concentration of 0.5 μM and incubated with cells for 30 min at 37°C. After incubation, cells were fixed in ice-cold, 100% methanol for 15 min and rinse 3 times with PBS, followed by immunostaining.

### Total RNA extraction and semi-quantitative real time RT-PCR

Cells were lysed in Trizol^®^ Reagent before one-fifth volume of chloroform was added. The mixture was then centrifuged at 13,000×rpm for 15 min at 4°C. The aqueous phase was then mixed with equal volume of 100% isopropanol and incubated for 10 min at room temperature RNA was precipitated by centrifugation at 13,000×rpm for 10 min at 4°C. RNA pellet was washed with 70% RNase-free ethanol and dissolved in RNase-free H_2_O.

Three micrograms of total RNA were used to perform reverse transcription using Expand reverse transcriptase (Roche, USA) at 42°C for 1 h in a total volume of 20 μl. Equal amount of cDNAs were then PCR-amplified using appropriate primers. The primer sequences for amplification of TNF-α are 5′-CCTCTCTCTAATCAGCCCTCTG-3′ (forward) and 5′-GAGGACCTGGGAGTAGATGAG-3′ (Reverse); the primer sequences for amplification of TRAIL are 5′-ACTTGAGGAATGGTGAAC-3′ (forward) and 5′-GGTCAGGATAACTTGTGTAT-3′(reverse); the primer sequences for amplification of FasL are 5′-GCAGCCCTTCAATTACCCAT-3′ (forward) and 5′-CAGAGGTTGGACAGGG AAGAA-3′ (reverse).

### Pharmacological inhibition experiment

For the NF-кB pathway inhibition experiment, HeLa cells were infected with NDV, followed by incubating with 5 μM IKK16 or DMSO. Cells were harvested at 16 and 20 h.p.i., and subjected to semi-quantitative real time RT-PCR, immunostaining, or Western blot analysis.

For the inhibition of caspase 8 or RIP1 activity, HeLa cells were infected with NDV, followed by the treatment of 40 μM Z-IEVD-FMK or 200 μM Necrostatin. Cells were harvested at 16 and 20 h.p.i., and subjected to Western blot analysis.

For the stimulation of necroptosis, HeLa cells were treated with 50 ng/ml TNF-α, 20 μM Z-VAD-FMK, and 2 μM AT406 (T+A+Z) for 10 h.

### Immunoprecipitation

Cells were washed with ice-cold PBS and lysed in RIPA buffer (50 mM Hepes, PH 7.4, 150 mM NaCl, 10 mM EDTA, 1% Triton X-100, 1 mM PMSF, 1 mMNa_3_VO_4_, 1mM NaF, 0.5% sodium deoxycholate) supplemented with Protease Inhibitor Cocktail (Biotool, B14001). The cell lysates were centrifuged at 12000 rpm for 20 min. Next, the supernatant was incubated with 2.5 μl anti-RIP1-N at 4°C overnight, followed by incubation with 70 μL of protein G-agarose for 2 h (Invitrogen, 15920-010). The beads were rinsed with washing buffer (50 mM Hepes, 50 mM Nacl, 10 mM EDTA, PH 7.4) thrice and 50 μl protein loading buffer was added. The immunoprecipitated proteins were subsequently analyzed with Western blotting using anti-RIP1-N, anti-phosphor-MLKL, and anti-MLKL.

### ELISA

Human TNF-α ELISA kit (E-EL-H0109) and human TRAIL (E-EL-H1593c) ELISA kit were purchased from Elabscience Biotechnology Co. Ltd. HeLa cells were infected with NDV for 12, 16, 20 h, and the supernatant were incubated with ELISA plate. After 1.5 h, supernatant was removed and the plate was incubated with biotinylated anti-TNF-α or anti-TRAIL for 1 h. Antibody was removed and the wells were washed for 3 times, followed by incubating with HRP-conjugate secondary antibody. After 5 times washing, plate was incubated with substrate reagent for 15 min, reaction was terminated by stop solution. Above experiment was performed at 37°C. Plate was read at 450 nm and result was calculated according to the standard curve.

### Virus titration

Cell-free supernatant of NDV-infected cells collected at different time points post-infection was clarified by centrifugation and 10-fold serially diluted using serum-free DMEM. The viral titers were determined by 50% infectious dose (TCID_50_) assay. Briefly, serially diluted aliquots of virus were applied to confluent monolayers of DF-1 cells in 96-well plates. After 2 h of absorption, unbound viruses were removed, and the cells were washed twice with DMEM. The plates were incubated with DMEM at 37°C and the cytopathic effect (CPE) was observed after 3 days. Each sample was titrated in triplicate. The tissue culture TCID_50_ is calculated using Reed and Munch mathematical analysis of the data.

### Plasmid construction

RIP1 were PCR amplified from HeLa cellular cDNAs and cloned into vector Flag14 between restrict enzyme XbaI and EcoRI under control of a cytomegalovirus promoter, generating Flag14-RIP1, with Flag-tag at C-terminus. Primers for PCR amplification are 5′-GACCGGAATTCTCCACCATGCAACCAGACATGTCCTTGAATG-3′ (forward) and 5′-GACCGTCTAGAGTTCTGGCTGACGTAAATC AAGCTGCTC-3′ (reverse).

PXJ40F-RIP1-D324K and PXJ40F-RIP1-K45A mutants were constructed by site-directed overlap two round PCR mutagenesis. Primers for D324K mutagenesis are 5′-TCTCTTCAACTTAAGTGTGTGGCAGTACCTT-3′ (forward) and 5′-AAGGTACTGCCACACACTTAAGTTGAAGAGA-3′ (reverse). Primers for K45A mutagenesis are 5′-GGGACTCATGATCATGGCCACAGTGTACAAG GG-3′ (forward) and 5′-CCCTTGTACACTGTGGCCATGATCATGAGTCCC-3′ (reverse).

## SUPPLEMENTARY MATERIALS FIGURES AND TABLES


