# Supplementary material for: RIP1 is a central signaling protein in regulation of TNF-α/TRAIL mediated apoptosis and necroptosis during Newcastle disease virus infection
Source: Oncotarget. 2017 May 18;8(26):43201–17. doi: 10.18632/oncotarget.17970 (PMC5522139; doi:10.18632/oncotarget.17970)
Supplement: Supplementary file 1 [file oncotarget-08-43201-s001.pdf]

RIP1 is a central signaling protein in regulation of TNF-α/TRAIL mediated apoptosis and necroptosis during Newcastle disease virus infection

SUPPLEMENTARY MATERIALS

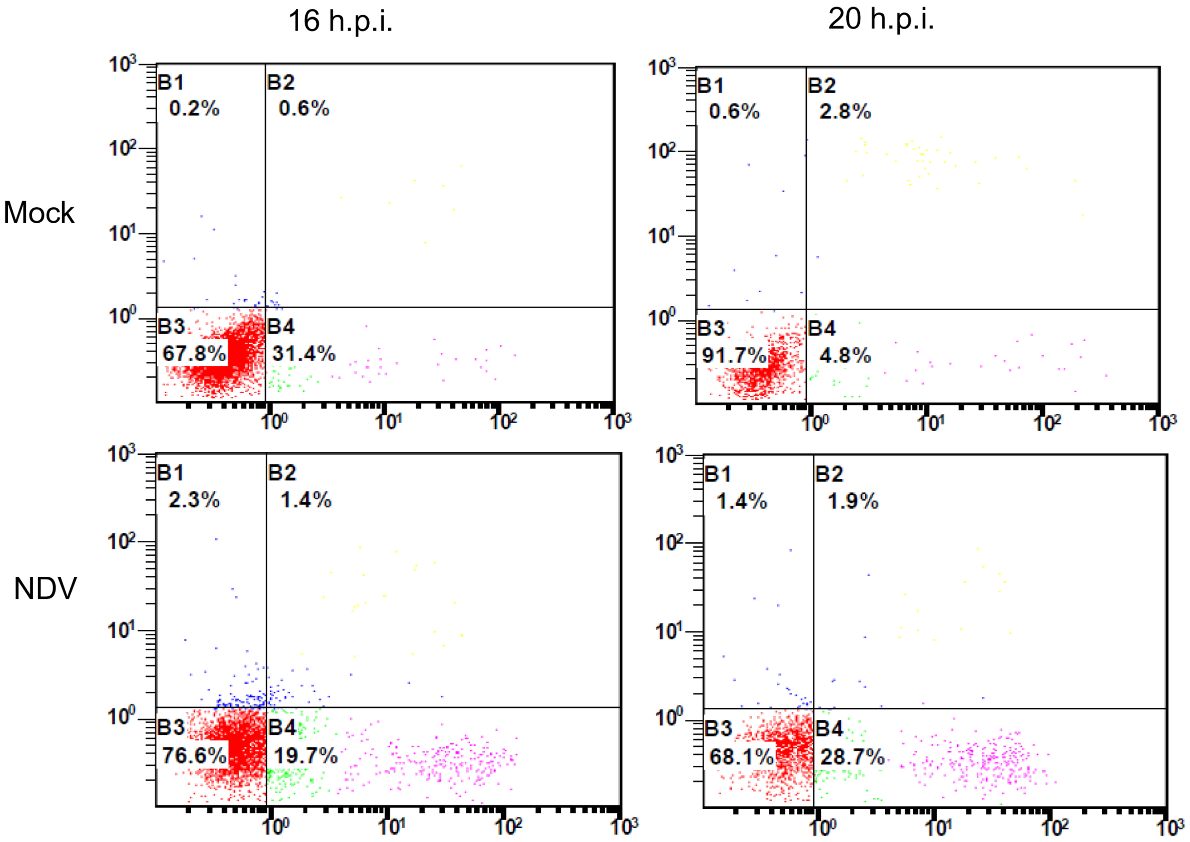

Supplementary Figure 1: NDV infection mainly induces apoptosis. HeLa cells were mock-infected or NDV-infected, and harvested at 16 and 20 h.p.i.. Cells were stained with annexin V-FITC and PI, subjected to FACS. Annexin V-FITC stains apoptotic cells and PI stains late apoptotic or necrotic cells. 19.7%-28.7% cells were stained with Annexin V-FITC from 16 to 20 h.p.i., undergoing early apoptosis. 1.4-1.9% cells were stained with both Annexin V-FITC and PI staining from 16 to 20 h.p.i., undergoing necroptosis or late apoptosis.

FACS: HeLa cells were infected with NDV, harvested at indicated time points, washed in cold PBS, and resuspended in 1× Annexin-binding buffer. 5 µL Alexa Fluor® 488 Annexin V and 1 µl PI (100 µg/ml) was added to each 100 µL of cell suspension and incubated at room temperature for 15 min. Subsequently, 400 µl 1× Annexin-binding buffer was added and mix gently. Samples were kept on ice, followed by flow cytometry to sort the staining cells. The fluorescence emission was examined at 530 nm and >575 nm.

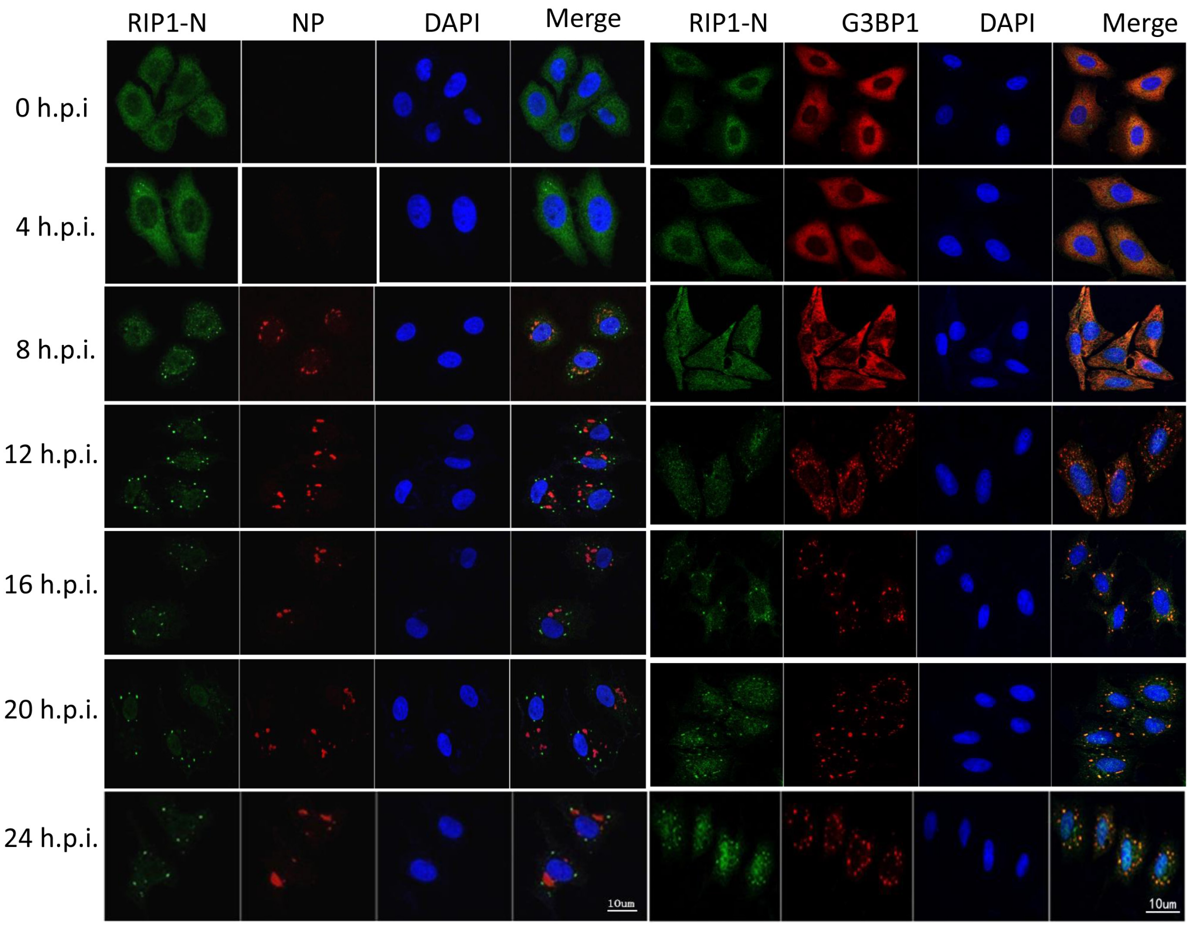

Supplementary Figure 2: RIP1 aggregates to NDV-induced SGs at 8-24 h.p.i.. HeLa cells were infected with NDV, subjected to immunostaining at 0, 4, 8, 12, 16, 20, 24 h.p.i.. The subcellular distribution of RIP1 (green) and NP or G3BP1 (red) were analyzed with anti-RIP1-N, anti-NP, and anti-G3BP1. Nuclei were stained with DAPI (blue). Merged images illustrate RIP1/NP/DAPI and RIP1/G3BP1/DAPI fluorescence.

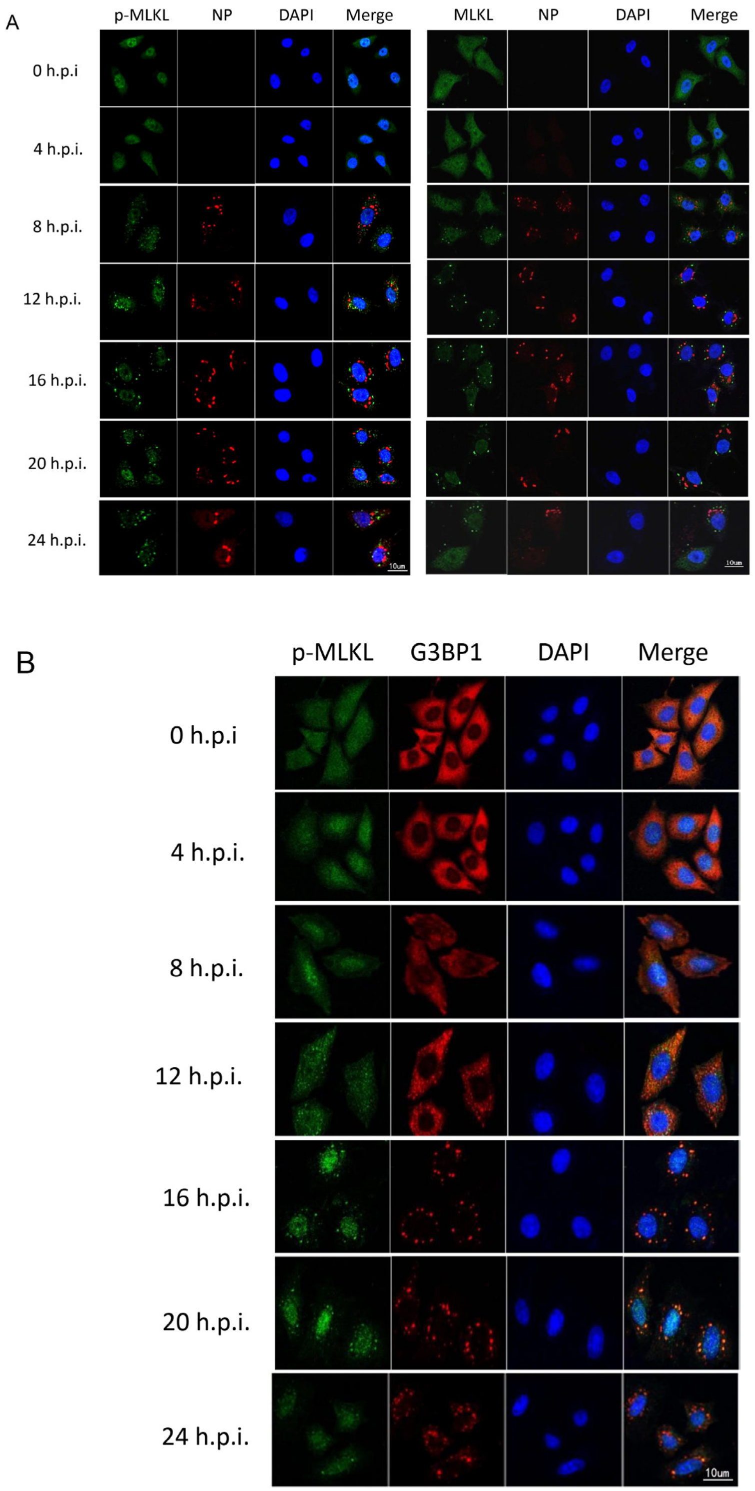

Supplementary Figure 3: Phosphor-MLKL and MLKL clusteres to SGs from 8 to 24 h.p.i. (A) HeLa cells were infected with NDV, and harvested at 0, 4, 8, 12, 16, 20, and 24 h.p.i.. The subcellular distribution of phosphor-MLKL or MLKL (green), NP (red) were analyzed with immunostaining using anti-phosphor-MLKL or MLKL, and anti-NP. Nuclei were stained with DAPI (blue). Merged images illustrate p-MLKL /NP/DAPI and MLKL/NP/DAPI fluorescence. **(B)** The subcellular distribution of phosphor-MLKL (green) and G3BP1 (red) were analyzed with immunostaining using anti-phosphor-MLKL and anti-G3BP1. Nuclei were stained with DAPI (blue). Merged images illustrate p-MLKL/G3BP1/DAPI fluorescence.

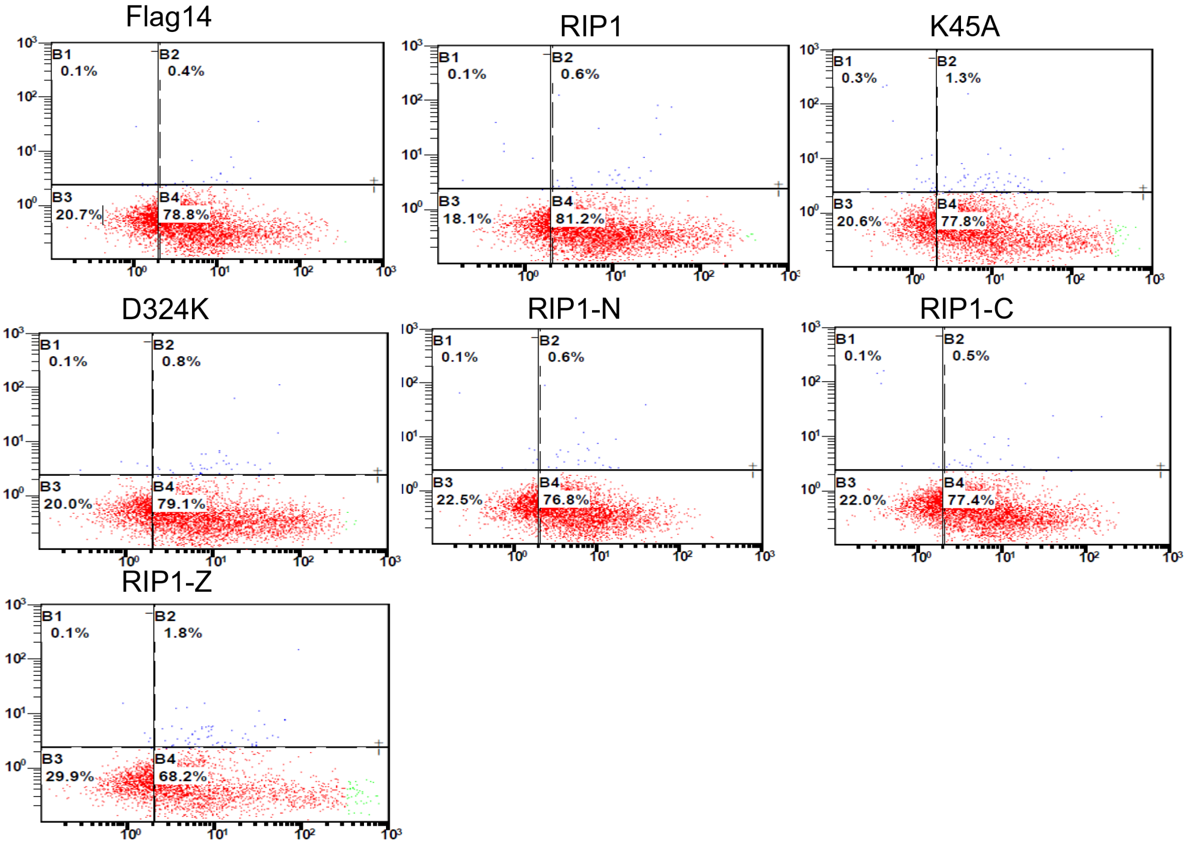

Supplementary Figure 4: NDV infection mainly induces apoptosis, not necroptosis. HeLa cells were transfected with plasmids Flag14, RIP1, RIP1-N, RIP1-C, RIP1-Z, K45A, D324K for 20 h, followed by NDV infection. Cells were harvested at 24 h.p.i., and subjected to flow cytometry using Annexin V-FITC and PI. (RIP1-N: 1-324 aa; RIP1-C: 325-671 aa, RIP1-Z: 496-583 aa).

Flag-RIP1-N, Flag-RIP1-C, and Flag-RIP1-Z were PCR amplified from Flag-RIP1 and cloned into vector Flag14 between restrict enzyme XbaI and EcoRI under control of a cytomegalovirus promoter, with Flag-tag at C-terminus. Primers for these plasmids are listed below: RIP1-N (forward): 5′-GACCGAAGCTTGCTTGCCACCATGCAACCAGACATGTCCTTGAATG-3′; RIP1-N (reverse): 5′-GACCGGGATCCATCAAGTTGAAGAGACTGCAGCATTCTCTTCA-3′; RIP1-C (forward): 5′-GACCGAAGCTTGCCACCATGTGTGTGGCAGTACCTTCAAGCCGGT-3′; RIP1-C (reverse): 5′-GACCGTCTAGAGTTCTGGCTGACGTAAATCAAGCTGCTC-3′; RIP1-Z (forward): 5′-GACCGAAGCTTGCCACCATGCAACCAGACATGTCCTTGAATG-3′; RIP1-Z(reverse): 5′-GACGCTCTAGAGTTCTGGCTGACGTAAATCAAGCTGCTC-3′.
